# Pandemic Puppies: Characterising Motivations and Behaviours of UK Owners Who Purchased Puppies during the 2020 COVID-19 Pandemic

**DOI:** 10.3390/ani11092500

**Published:** 2021-08-25

**Authors:** Rowena M. A. Packer, Claire L. Brand, Zoe Belshaw, Camilla L. Pegram, Kim B. Stevens, Dan G. O’Neill

**Affiliations:** 1 Department of Clinical Sciences and Services, The Royal Veterinary College, Hawkshead Lane, North Mymms, Hatfield, Hertfordshire AL9 7TA, UK; clbrand@rvc.ac.uk; 2 EviVet Evidence-Based Veterinary Consultancy, Nottingham NG2 5HU, UK; z.belshaw.97@cantab.net; 3 Department of Pathobiology and Population Sciences, The Royal Veterinary College, Hawkshead Lane, North Mymms, Hatfield, Hertfordshire AL9 7TA, UK; cpegram@rvc.ac.uk (C.L.P.); kstevens@rvc.ac.uk (K.B.S.); doneill@rvc.ac.uk (D.G.O.)

**Keywords:** dogs, puppy, COVID-19, lockdown, welfare, human-animal interaction

## Abstract

**Simple Summary:**

Widespread media reports suggest that unusually high numbers of the public purchased, or sought to purchase, puppies following the first ‘lockdown’ phase of the COVID-19 pandemic in the UK. This study aimed to explore this phenomenon by comparing the reasons why, the methods how, and by whom “Pandemic Puppies” were purchased during this period (from 23 March 2020–31 December 2020), and compare these responses with owners who purchased their dog during the same date-period in 2019. Valid responses were analysed from owners of 1148 puppies from 2019 and 4369 Pandemic Puppies. Key differences included Pandemic Puppy owners being more likely to be first-time dog owners, have children in their household, pay a deposit without seeing their puppy, collect their puppy from outside their breeders’ property, see their puppy without their littermates, and pay > £2000 for their puppy, compared with 2019 puppies. Over 1 in 10 Pandemic Puppy owners had not considered purchasing a puppy before the pandemic, while 2 in 5 felt their decision to purchase a puppy had been influenced by the pandemic, most commonly due to having more time to care for a dog. Changes in puppy purchasing during the pandemic raise a range of welfare concerns including relinquishment, behavioural problems and poor health.

**Abstract:**

Widespread media reports suggest that unusually high numbers of the public purchased, or sought to purchase, puppies following the first ‘lockdown’ phase of the COVID-19 pandemic in the UK, dubbed “Pandemic Puppies”. This study aimed to explore this phenomenon by comparing pre-purchase motivations and behaviours, and purchase behaviours of UK owners purchasing puppies aged < 16 weeks from 23 March 2020–31 December 2020 with those of owners who purchased puppies during the same date-period in 2019. An online survey was conducted during November-December 2020, from which 5517 valid responses were analysed (2019 puppies: *n* = 1148; 2020 ‘Pandemic Puppies’: *n* = 4369). Over 1 in 10 Pandemic Puppy owners had not considered purchasing a puppy before the pandemic, and 2 in 5 felt their decision to purchase a puppy had been influenced by the pandemic, most commonly by having more time to care for a dog (86.7%). Multivariable logistic regression models revealed that Pandemic Puppy owners were more likely to be first-time dog owners and have children in their household, were less likely to seek out a breeder that performed health testing on their breeding dog(s) or view their puppy in-person, and were more likely to pay a deposit without seeing their puppy. At purchase, Pandemic Puppies were more likely to be younger, delivered or collected from outside their breeders’ property, seen without their littermates, and cost > £2000 compared with 2019 puppies. Changes in puppy purchasing during the pandemic raise welfare concerns for this unique population, including relinquishment, behavioural problems and poor health.

## 1. Introduction

**Figure 1:**
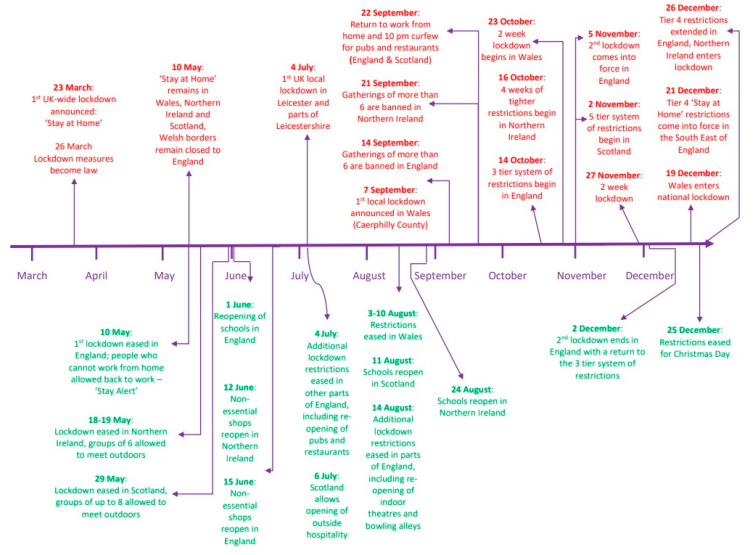
Timeline of the introduction and easing of COVID-19 restrictions in the UK during 2020. The UK entered lockdown on 23 March 2020. Following this the devolved nations (Northern Ireland, Wales and Scotland) followed their own paths in determining restrictions and, in turn, easing restrictions. The events shown here are not exhaustive but serve to demonstrate the main periods of social restrictions upon residents of the UK from 23 March–31 December 2020.

Efforts to suppress the spread of the SARS-CoV-2 coronavirus in the UK during 2020 involved periods of national and local lockdown along with other restrictions that changed the lifestyles of UK households (see Figure 1). These periods of variable confinement, whereby a large proportion of the public spent several months restricted at home with limited external social contact, commenced on 23 March 2020 with a ‘stay at home’ order across the whole of the UK [1]. During April to May 2020, media and Non-Governmental Organisation (NGO) reports, began to emerge suggesting that unusually high numbers of the UK population had sought to purchase a puppy, dubbed the “Pandemic Puppy” phenomenon [2]. The true extent of puppy purchasing is challenging to quantify in the UK owing to the largely ‘cottage industry’ nature of dog breeding and selling, and lack of a national dog licensing scheme. However, as a proxy, data on activity on puppy-finding websites supported a marked increase in puppy purchasing intention. The Kennel Club (KC) reported that searches using its “Find a Puppy” online tool increased by 168% between 23 March and 31 May 2020 compared to the same period in 2019, with May 2020 alone accounting for a 237% increase during a period when the majority of UK residents continued to work from home [3,4]. Similarly, the Dogs Trust reported that online searches regarding puppy acquisition increased by 120% in April 2020 [5] whilst statistics from the People’s Dispensary for Sick Animals (PDSA) and the Brachycephalic Working Group (BWG) demonstrated that internet searches via Google for ‘buying a puppy’ increased by 175% within one month of the UK lockdown [4,6]. Rising demand for dog ownership in April and May 2020 has been reported internationally [7,8].

This ‘Pandemic Puppy’ phenomenon has given rise to concerns that high demand for puppies during this period may have exhausted supply from good welfare sources and thereby pushed prospective puppy buyers towards purchasing from unscrupulous breeders and sellers hoping to ‘cash in’ on the phenomenon by producing large numbers of puppies under low welfare conditions, including puppy farms and via illegal importation at inflated prices [4]. In addition, concerns were raised over a rising risk of impulse purchasing of puppies, particularly by households unsuited to maintaining an appropriate human lifestyle for dog ownership after lockdown restrictions were lifted [2]. Impulse buying, defined as unplanned behaviour involving quick decision-making and a tendency toward immediate acquisition of a product [9], was observed for a variety of products perceived to be scarce during the COVID-19 pandemic [10]. Impulse purchasing of puppies pre-dates the pandemic, with the power of the desire to ‘own a puppy right now’ eclipsing rational human thought, with the inherent “cuteness” of puppies at weaning age [11] considered a facilitator of such purchases [12]. Impulsive purchasing of puppies during the pandemic has been reported [2], but has not been evidenced by scientific study and comparison with impulsivity of puppy purchases prior to the pandemic.

Research has demonstrated that many aspects of the puppy-purchasing process, from decision-making through the actual purchasing behaviour, were already suboptimal in many respects in the UK prior to the pandemic [13]. In one UK study (*n* = 1844), one quarter (461/1844) of puppies were acquired under eight weeks of age and 8.1% were obtained without viewing their mother (dam) [14]. In a further study of UK dog owners (*n* = 1427), one sixth (15.7%) of owners admitted that they had carried out no pre-purchase research, one quarter of owners purchased their puppy on first visit (24.7%), and nearly half (46.1%) of owners did not ask to see health records for either the dam or sire of their puppy [15]. Such sub-optimal purchasing behaviours leave owners vulnerable to purchases from unscrupulous breeders and suppliers, including illegal imports and puppy farms [16], who breed to meet demand rather than good health or welfare, and which can have long-term negative consequences for behaviour and welfare in these dogs [17,18,19,20].

A plethora of studies on the impact of COVID-19 on canine welfare have been published during 2020 and 2021; however, these papers have largely focused upon dogs that were purchased and owned pre-pandemic. Specific topics have included dog-owner relationships [8,21,22,23,24,25], the social support provided by dogs to owners [22,24,25,26,27,28,29], owner concerns over caring for their dog and accessing veterinary services [21,22,23,28,29,30,31], adoption from and relinquishment to rescue centres [8], along with changes in dogs routines and behaviour [23,29,32,33]. Thus, despite widespread interest and concern over the Pandemic Puppy phenomenon, there is a critical data gap on puppy purchasing during the pandemic.

The present study aimed to explore the impact of the COVID-19 pandemic upon puppy purchasing in the UK. Using a cross-sectional analysis of a national survey, we sought to:

(i) describe the pre-purchase motivations and behaviours and purchase behaviours of owners of puppies purchased 23 March–31 December 2020;
(ii) compare data from (i) with the motivations and behaviours of owners of puppies purchased during the same date frame in 2019.

## 2. Materials and Methods

### 2.1. Survey Design and Content

**Figure 2:**
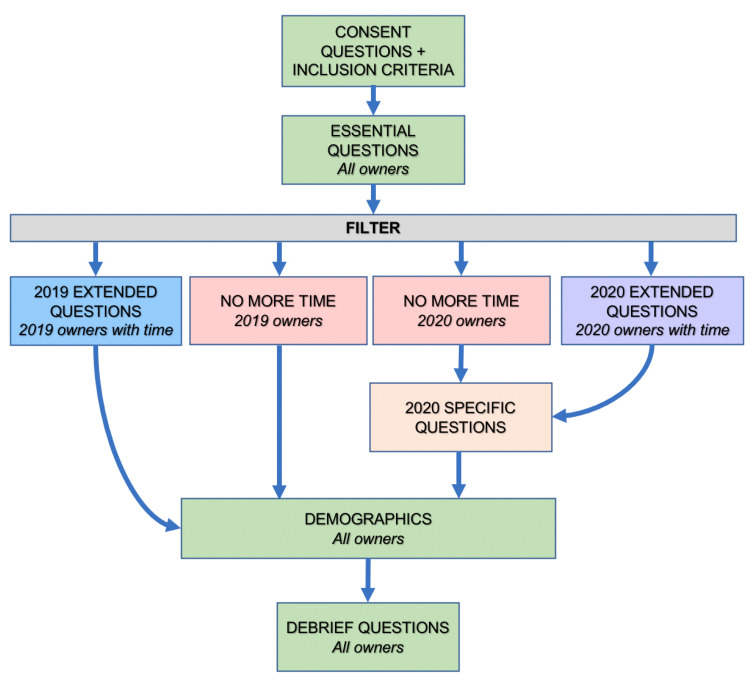
Schematic representation of the survey logic employed to allow different survey routes for owners depending upon year of acquisition and time constraints.

An online questionnaire was designed to explore the pre-purchase and purchase motivations and behaviours of UK puppy purchasers. Questions were designed iteratively amongst the authors and piloted on a small number of respondents to ensure ease of understanding and comprehensiveness of scope. The survey included five broad sections: (1) General owner demographics e.g., gender, age, household members, prior dog ownership; (2) General puppy demographics e.g., breed, sex; (3) Pre-purchase motivations e.g., factors influencing choice of breed; (4) Pre-purchase behaviours e.g., research conducted; (5) Purchase behaviours e.g., requests for health records, cost of puppy, which parents of their puppy were seen (if any). Owners of Pandemic Puppies were additionally directed to a set of questions exploring COVID-specific impacts. This study received ethical approval from the Social Science Research Ethical Review Board at the Royal Veterinary College (URN: SR2020-0259). To reduce missing data for core study questions, ‘survey logic’ was used to offer every owner the option to complete just core questions, or core questions plus an extended set of questions (Figure 2). The full survey is included in Supplementary Material S1 File.

### 2.2. Participant Recruitment

The questionnaire was hosted on SurveyMonkey and was open from 10 November to 31 December 2020. The survey was distributed by snowball sampling via a wide range of sources, including social media, the veterinary, canine, and general press (including radio interviews and printed articles), and through key stakeholders including the commercial sector (e.g., insurance bodies, canine registration organisations and puppy-selling websites) and charity sector (e.g., animal welfare and rehoming charities).

Respondents were required to give informed consent for their data to be held on a secure server in accordance with UK GDPR legislation. IP addresses were used to eliminate duplicate responses prior to permanent deletion.

#### Inclusion Criteria

Respondents were required to:

1. Be over 18 years of age
2. Resident in the UK
3. Have brought home a puppy aged under 16 weeks at any date during 2019 or 2020 (N.B. although all puppy purchases in 2019–2020 were eligible for the broader project, responses analysed in this study were limited to owners who brought home their puppy between 23 March–31 December during 2019 or 2020).
4. Have purchased their puppy rather than rehomed or bred the puppy themselves.

Where participants had purchased more than one puppy during the period, they were asked to answer for the youngest at the time of the survey and, in the case of co-purchased littermates, owners were asked to answer for the dog whose name came first alphabetically.

### 2.3. Data Cleaning

The raw survey data were exported from SurveyMonkey into Microsoft Excel for manual data cleaning prior to analysis, including removing responses from duplicated IP addresses (where the more complete response was retained), responses with no data beyond the consent and inclusion criteria stage, and respondents that did not meet inclusion criteria.

### 2.4. Spatial Analysis

Participants were asked to provide the first half of their postcode to allow assessment of the representativeness of the study sample to the UK population. These partial postcode data were checked for validity against the Office for National Statistics (ONS) National Statistics Postcode Lookup (NSPL) May 2021 data [34] and were allocated to one of the 12 UK regions. Regional response rates were calculated as (total responses from a region in a year/ONS region population [35]) ×100,000. Choropleth maps were produced using ArcGIS 10.2 (Environmental Systems Research Institute, Redlands, California, CA, USA) to show the regional response rates per 100,000 population for 2019 and 2020, together with the percentage increase in response rate between the two years.

### 2.5. Qualitative Content Analysis of Free-Text Options

In addition to fixed-choice responses to multiple-choice questions, many questions included an ‘Other, please specify’ answer to capture novel owner insights. To allow these responses to be quantified alongside existing fixed-choice responses, free-text data were coded using qualitative content analysis [36]. Three authors (R.M.A.P., Z.B. and C.L.B.) familiarised themselves with the data by reading all free-text responses to the multiple-choice option ‘Other, please specify’. Two of these authors (Z.B. and C.L.B.) independently used an inductive approach similar to that explained elsewhere to develop a coding framework for each question [37]. Based on the two sets of independently derived codes, Z.B. and C.L.B. agreed an overall set of categories that were finalised by R.M.A.P. and applied to the free-text responses by C.L.B., C.L.P. and R.M.A.P. Where free-text responses were deemed to fit within the scope of existing fixed-choice responses, considered to be deductive categories, data were back allocated into that category if not already selected. The inductive coding framework for each question including free-text responses can be found in Supplementary Material S2 File.

### 2.6. Quantitative Analysis

Following cleaning in Excel, data were imported into IBM SPSS Statistics v27 (SPSS Inc, Chicago, IL, USA). Initial data analysis included calculation of descriptive statistics (frequency and percentage) for all variables. Data from puppies purchased between 23 March–31 December 2019 (“2019 puppies”) and puppies purchased between 23 March–31 December 2020 (“Pandemic Puppies”) were compared at the univariable level using chi-squared (*X*^2^) analysis for categorical variables and Mann-Whitney U tests for non-normally distributed continuous data (with data distribution ascertained by visual inspection of histograms). Variables liberally associated with acquisition year in the univariable analyses (*p* < 0.2) were included in four multivariable binary logistic regression models describing (i) demographics, (ii) pre-purchase motivations, (iii) pre-purchase behaviour, and (iv) purchase behaviour, with year of acquisition (2019 vs. 2020) as the binary outcome. Model development used manual backwards stepwise elimination. Confounding was assessed for all variables retained in the final models through addition of each independent variable in a stepwise manner to the model and assessing for substantial (>20%) change in OR of any other variable when each new variable was added to the model [38]. Collinearity of variables was assessed through evaluation of the correlation matrices, the variance inflation factor (VIF) and tolerance [39]. Interactions between all independent variables in the final models were assessed for significance. The Hosmer-Lemeshow test was used to evaluate the quality of the model fit. Statistical significance was set at the 5% level.

## 3. Results

In total, *n* = 7545 responses were returned to the survey. Following cleaning, *n* = 96 responses were removed due to duplicated IP addresses, *n* = 799 responses were removed that held no data beyond the consent and inclusion criteria, *n* = 123 were removed due to not meeting inclusion criteria (of which *n* = 53 had brought their puppy home after the age of 16 weeks, *n* = 30 had purchased their puppy from another owner choosing to relinquish their puppy, *n* = 25 had adopted their puppy rather than purchased it, *n* = 8 who were yet to bring their puppy home, *n* = 5 had bred the puppy themselves, *n* = 1 who had purchased their puppy from a third-party seller, and *n* = 1 whose puppy was part of a ‘puppy walking scheme’ for an assistance dog charity). Of the remaining valid sample (*n* = 5517), there were 1148 puppies (20.8%) purchased between 23 March–31 December 2019 (“2019 puppies”) and 4369 puppies (79.2%) purchased between 23 March–31 December 2020 (“Pandemic Puppies”).

### 3.1. Spatial Analysis

**Figure 3:**
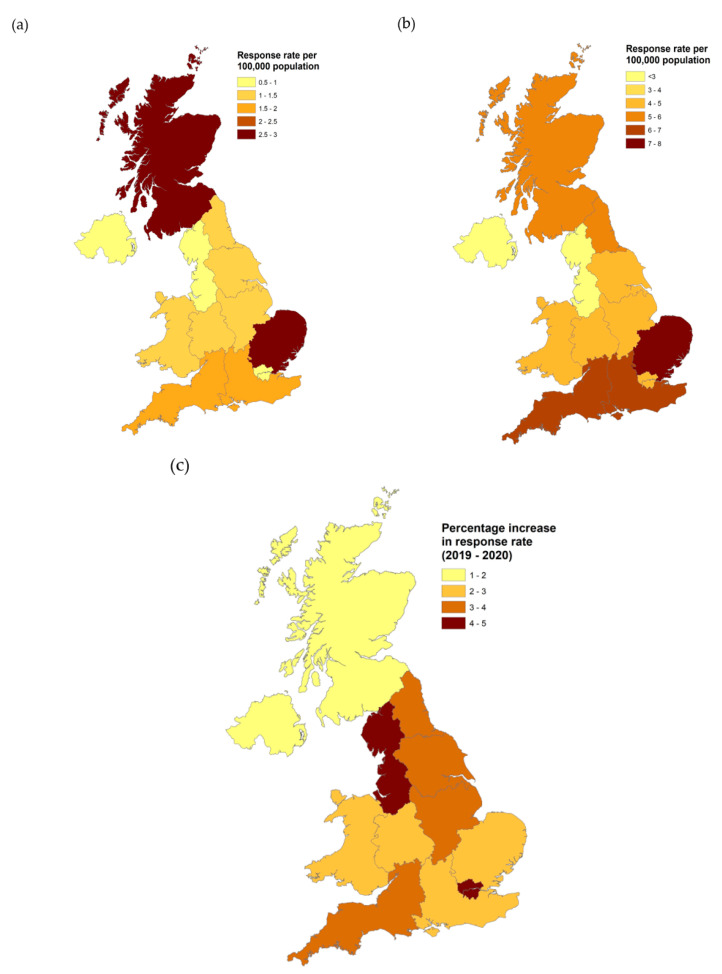
Choropleth maps of the UK describing the distribution of respondents who gave postcode data. (**a**) Respondents per 100,000 of UK population 2019 (*n* = 946); (**b**) Respondents per 100,000 of UK population 2020 (*n* = 3507); (**c**) percentage change of response rates between 2019 and 2020.

All UK regions were represented in the sample, with geographical distribution of respondents by year and changes between years described in Figure 3. There was no significant difference in geographical distribution of the sample compared with Office for National Statistics population data for mid-2020 (*t* = 0.013, df = 11, *p* = 0.990). Owners of Pandemic Puppies were significantly less likely to live in Scotland (2019: 15.8% vs. 2020: 9.2%) and more likely to live in London (2019: 6.8% vs. 2020: 20.7%; *X*^2^ = 64.41, *p* < 0.001) than 2019 puppies.

### 3.2. Owner Demographics and Lifestyle

Owners were predominantly female, with no difference in gender distribution between 2019 puppy and Pandemic Puppy owners (female, 2019: 92.0% vs. 2020: 90.0%; *X*^2^ = 6.61, *p* = 0.202). The most common age group of owners was 45–54 years old (2019: 25.2% vs. 2020: 24.6%) followed by 25–34 years (2019: 23.4% vs. 2020: 24.1%), with no difference in age between Pandemic Puppy and 2019 puppy owners (*X*^2^ = 11.77, *p* = 0.067). The majority of owners were the primary carer for their puppy (i.e., providing the majority of care such as feeding and walking; 2019: 62.9% vs. 2020: 57.8%); however, Pandemic Puppy owners were significantly more likely to share the caring role with someone else in their household (2019: 34.1% vs. 2020: 39.7%; *X*^2^ = 11.28, *p* = 0.024).

#### 3.2.1. Experience with Dogs

Pandemic Puppy owners were less likely to have previously owned a dog, with 59.7% of Pandemic Puppy owners having previous dog ownership experience compared with 66.7% of 2019 puppy owners (*X*^2^ = 16.90, df = 2, *p* < 0.001). There was no difference in proportion between owners of Pandemic Puppies and 2019 puppies that had grown up with a dog (2019: 68.1% vs. 2020: 69.3%, respectively; *X*^2^ = 0.48, df = 1, *p* = 0.487). Owners of Pandemic Puppies were less likely to be employed in the canine and/or animal care sector (e.g., veterinary surgeons, veterinary nurses or dog trainers) compared with 2019 puppy owners (2019: 17.9% vs. 2020: 10.0%; *X*^2^ = 49.02, df = 2, *p* < 0.001).

#### 3.2.2. Household Demographics

Demographics of Pandemic Puppy households differed in several ways to those of 2019 puppy households at the univariable level. Pandemic Puppies were more likely to live in households with both adults and children compared to 2019 puppy owners (2019: 26.8% vs. 2020: 33.2%) (*X*^2^ = 15.64, df = 3, *p* < 0.001). Of those households with children, children in Pandemic Puppy households were more likely to be younger, with half aged 5–10 years (2019: 37.8% vs. 2020: 50.3%; *X*^2^ = 14.69, *p* < 0.001), and less likely to be older teenagers aged 16–18 years (2019: 35.4% vs. 2020: 24.8%; *X*^2^ = 13.69, *p* < 0.001). Pandemic Puppies were more likely to be the only dog in the household than 2019 puppies, with 70.1% of Pandemic Puppies living in single-dog households vs. 58.7% of 2019 puppies (*X*^2^ = 54.62, df = 3, *p* < 0.001). The majority of Pandemic Puppies lived in households with access to a private garden or yard (95.1%), which did not differ from 2019 puppies (96.9%; *X*^2^ = 7.78, df = 3, *p* = 0.051).

#### 3.2.3. Impact of COVID on Household Lifestyle

At the univariable level, during the 2020 phase of the pandemic, Pandemic Puppy owners were more likely to have worked from home (2019: 51.8% vs. 2020: 57.3%; *X*^2^ = 10.01, df = 1, *p* = 0.002) and to have been home-schooling children (2019: 22.7% vs. 2020: 27.5%; *X*^2^ = 9.11, df = 1, *p* = 0.003) compared with owners’ of 2019 puppies. In contrast, during the 2020 phase of the pandemic, significantly fewer owners of Pandemic Puppies had been furloughed from their job compared with owners’ of 2019 puppies (2019: 30.7% vs. 2020: 24.6%, respectively; *X*^2^ = 15.63, df = 1, *p* < 0.001). There was no difference in the proportion of owners who became unemployed due to COVID-19 between Pandemic Puppy owners and 2019 puppy owners (2019: 5.8% vs. 2020: 6.0%; *X*^2^ = 0.07, df = 1, *p* = 0.794), in the proportion considered to be keyworkers (2019: 34.8% vs. 2020: 33.7%; *X*^2^ = 0.43, df = 1, *p* = 0.513), or to have another household member considered to be a keyworker (2019: 24.8% vs. 2020: 24.3%; *X*^2^ = 0.10, df = 1, *p* = 0.748).

### 3.3. Puppy Demographics

Detailed breakdown of puppy demographics will be provided in a follow-on publication. For context, the majority of puppies in the overall study were purebred (72.0%) (i.e., of a recognised breed). The proportion of purebred puppies in the 2020 Pandemic Puppies population was lower than for the 2019 puppies population (2019: 78.7% vs. 2020: 70.3%; *X*^2^ = 32.484, df = 1, *p* < 0.001), with a corresponding increase in designer crossbred puppies in the Pandemic Puppies population (2019: 18.8% vs. 2020: 26.1%; *X*^2^ = 27.668, df = 1, *p* < 0.001). There was a significant reduction in puppies registered with The Kennel Club in the Pandemic Puppy population compared to the 2019 puppies population (2019: 58.2% vs. 2020: 46.2%; *X*^2^ = 54.1, *p* < 0.001). There was no difference in sex distribution between the 2019 puppies and Pandemic Puppies (male, 2019: 51.7% vs. 2020: 53.4%, *X*^2^ = 1.12, df = 1, *p* = 0.290).

### 3.4. Owner Demographics and Lifestyle: Multivariable Analysis

Multivariable logistic regression modelling identified four variables related to owner demographics and lifestyle with significant association with Pandemic Puppies (Table 1). The Hosmer-Lemeshow test indicated acceptable model fit (*p* = 0.904).

### 3.5. Pre-Purchase Motivations

Companionship for the owner was the most common reason cited as to why prospective owners wanted to purchase a dog in both 2019 Puppy and Pandemic Puppy owners (Table 2). Pandemic Puppy owners were significantly more likely to cite exercise encouragement, improving their/their family’s mental health and companionship for their children as reasons their household wanted to acquire a dog compared with 2019 owners (Table 2). Conversely, a significantly lower proportion of Pandemic Puppy owners cited companionship for their other dog(s), a specific working role (e.g., gundog) or a non-working role (e.g., dog sports) compared with 2019 owners. Puppy acquisition was less likely to be driven solely by the responding owner in the Pandemic Puppy population (2019: 46.6% vs. 2020: 41.4%, *X*^2^ = 9.26, *p* = 0.002), with a household-wide equal desire to acquire a puppy more common in the Pandemic Puppy population than in the 2019 puppy population (2019: 41.3% vs. 2020: 45.6%, *X*^2^ = 5.36, *p* = 0.021).

When deciding which breed or crossbreed to purchase, the most commonly cited characteristics sought by owners were being a good companion (most common reason in 2019 and 2020) and size being suited to owner lifestyle (second most common reason in 2019 and 2020). At the univariable level, Pandemic Puppy owners were significantly more likely to seek a breed/crossbreed whose size suited to their lifestyle, that they believed was good with children, was easy to train, that friends or family members currently owned and that was hypoallergenic, compared with 2019 puppy owners (Table 3). Conversely, 2019 puppy owners were significantly more likely to seek a puppy from a breed/crossbreed they had owned before or had working abilities or, conversely, had no specific characteristics in mind when looking for a breed/crossbreed, compared with Pandemic Puppy owners (Table 3).

When considering the characteristics that owners sought out in a breeder, the most common characteristics cited were consistent between 2019 and 2020, namely a breeder who would allow them to see the puppies’ mother (dam), who they felt cared for their dog, and that they felt was trustworthy. At the univariable level, Pandemic Puppy owners were significantly more likely to seek out a breeder they felt cared for their dogs and who communicated well with them, but were significantly less likely to seek out a breeder that performed health tests for the breed/crossbreed they wanted, who was a member of The Kennel Club Assured Breeders Scheme, or bred from dogs that had been awarded prizes at dog shows, compared with 2019 puppy owners (Table 4).

#### Pre-Purchase Motivations: Multivariable Analysis

Multivariable logistic regression modelling identified eleven variables related to pre-purchase motivations with significant association with Pandemic Puppies (Table 5). The Hosmer-Lemeshow test indicated acceptable model fit (*p* = 0.939).

### 3.6. Pre-Purchase Behaviours

Pre-purchase research was more commonly carried out by Pandemic Puppy owners (+11.4%) and was reported by 58.1% of Pandemic Puppy owners compared with 46.7% of 2019 puppy owners. However, this difference was in part explained by the proportion of owners in each cohort who considered themselves to be an experienced dog owner who did not need to conduct pre-purchase research, which was 11.4% higher in 2019 puppy owners (50.3%) than Pandemic Puppy owners (38.9%) (*X*^2^ = 49.76, df = 2, *p* < 0.001). Only 3.0% of owners in each year reported that they conducted no pre-purchase research. Of those owners who conducted pre-purchase research, the most common sources were friends or family who own or had owned a dog (most common source in 2020 and joint most common source in 2019), alongside breed/crossbreed specific online resources (e.g., website/forum) and The Kennel Club website (second most common source in 2020). At the univariable level, Pandemic Puppy owners were significantly more likely to conduct pre-purchase research by talking to friends or family who own or had owned a dog before, using breed/crossbreed-specific online resources (e.g., forums, websites), using animal charity websites and reading books than 2019 puppy owners (Table 6).

Owners found their puppy most commonly via animal selling websites in 2019 and 2020, followed by their puppy’s breeder being somebody they already knew, such as a colleague, friend/family or someone they had bought a puppy from before. At the univariable level, Pandemic Puppy owners were more likely to find their puppy via animal selling websites or via breeder recommendations from friends, and significantly less likely to find their puppy by already knowing their puppy’s breeder, via The Kennel Club website, or from a physical advertisement compared to 2019 owners (Table 7).

**Figure 4:**
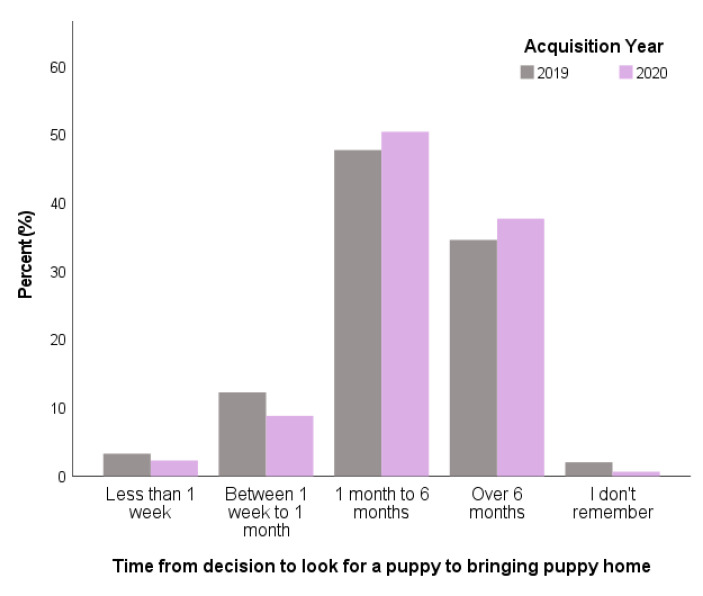
Interval from deciding to look for a puppy to acquisition (bringing puppy home) (*n* = 5401) with comparison between 2019 owners (*n* = 1124) and Pandemic Puppy owners (*n* = 4277) in the UK.

The interval from prospective owners’ initial decision to look for a puppy to when their puppy was brought home was most commonly between 1–6 months for both 2019 and 2020. At the univariable level, Pandemic Puppy owners were more likely to take longer from their decision to look for a puppy to acquiring their puppy than 2019 puppy owners, although this difference was small (*X*^2^ = 36.28, df = 4, *p* < 0.001; Figure 4). Around three-quarters of owners did not join a waiting list for their puppy in either year (2019: 73.0% vs. 2020: 71.5%) with no difference in levels between Pandemic and 2019 puppy owners (*X*^2^ = 0.76, df = 3, *p* = 0.683).

**Figure 5:**
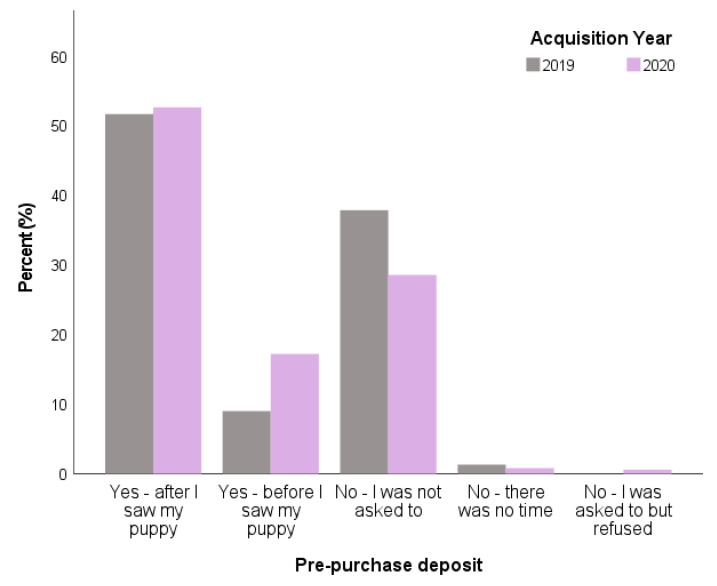
Deposit practices to secure puppies prior to purchase (*n* = 4463) with comparison between 2019 owners (*n* = 921) and Pandemic Puppy owners (*n* = 3452) in the UK.

At the univariable level, Pandemic Puppy owners were more likely to put down a deposit before they saw their puppy (2019: 8.9% vs. 2020: 17.2%; *X*^2^ = 81.87, df = 5, *p* < 0.001), and less likely to never be asked to put down a deposit for their puppy at any point during the puppy-purchasing process (2019: 37.5% vs. 2020: 28.6%) (Figure 5).

The majority of owners visited what was reported to be their puppies’ breeders house in-person prior to the day they brought the puppy home; however, this was significantly lower (−21.0%) in Pandemic Puppy owners compared to 2019 puppy owners (2019: 80.6% vs. 2020: 59.6%; *X*^2^ = 165.46, *p* < 0.001), with a corresponding significant increase (+22.4%) in owners using live video calls with their breeder (2019: 6.5% vs. 2020: 28.9%; *X*^2^ = 233.73, *p* < 0.001) or in breeders using photos/pre-recorded videos of what was claimed to be their puppy (+20.2% increase) (2019: 31.3% vs. 2020: 51.5%; *X*^2^ = 141.34, *p* < 0.001) (Table 8). The median (25th–75th centile) number of owner visits to see their puppy in-person prior to being brought home was 2 (1–3) visits for 2019 puppies, but was significantly lower for Pandemic Puppies at 1 (1–2) visit (U = −14.88, *p* < 0.001). Correspondingly, the median (25th–75th centile) number of live video calls with the breeder of Pandemic Puppies was significantly higher at 0 (0–3) calls compared to 0 (0–0) calls for 2019 puppies (U = 14.94, *p* < 0.001). One quarter of owners were not questioned on their suitability as a dog owner before their breeder agreed to sell them their puppy (2019: 75.6% vs. 2020: 75.2%); however, 2019 owners were less likely to remember whether the breeder asked them this question (“I don’t remember”, 2019: 4.3% vs. 2020: 2.7%; *X*^2^ = 9.24, df = 2, *p* = 0.011).

The majority of owners purchased their first choice of breed; however, owners of Pandemic Puppies were less likely to purchase their first-choice breed (2019: 91.6% vs. 2020: 86.6%; *X*^2^ = 42.09, df = 10, *p* < 0.001), with the most common reasons being that their first-choice breed was too expensive or they were unable to find a seller that had puppies available at the desired time of purchase (Table 9).

#### Pre-Purchase Behaviours: Multivariable Analysis

Multivariable logistic regression modelling identified eight variables related to pre-purchase behaviour with significant association with Pandemic Puppies (Table 10). The Hosmer-Lemeshow test indicated acceptable model fit (*p* = 0.689).

### 3.7. Purchase Behaviours

The most common location for owners to collect their puppy was the inside of what was reported to be the breeders home; however, at the univariable level there was a significant decrease (−33.7%) in this behaviour between 2019 and 2020 (2019: 84.7% vs. 2020: 51.0%; *X*^2^ = 407.7, *p* < 0.001), with a corresponding 24.3% increase in puppies being collected from outside their breeder’s home, for example their doorstep or garden (2019: 5.5% vs. 2020: 29.8%; *X*^2^ = 277.0, *p* < 0.001). Although rare, at the univariable level, Pandemic Puppies were significantly more likely to be delivered to their owners property, or collected from a service station or car park, compared with 2019 owners (Table 11). The vast majority of 2019 puppy (96.6%) and Pandemic Puppy (96.5%) owners were happy with the location they collected their puppy from, which did not significantly differ between years (*X*^2^ = 0.035, df = 1, *p* = 0.851).

The proportion of owners not asking their breeder to see any information related to the health testing related to their puppy’s parents increased significantly between 2019 and 2020, for both DNA (genetic) tests (+5.7% not asking; *X*^2^ = 20.90, df = 3, *p* < 0.001) and veterinary screening tests (e.g., hips, elbows, knees, eyes, respiratory testing; +10.4% not asking *X*^2^ = 42.19, df = 3, *p* < 0.001) at the univariable level (Table 12).

On the day that the puppies were finally acquired, puppies were most likely to be seen with their mother (dam) followed by their littermates); however, this was significantly lower for Pandemic Puppies, with over a quarter seen without their littermates (27.9%, −12.8% less than 2019 puppies) (Table 13), and one quarter seen without their dam (24.9%, −10.6% less than 2019 puppies). Pandemic Puppies were also significantly less likely to be seen with another dog(s) they were not related to, their sire, other adult dog(s) they were related to, or other puppies (that the owner was unsure whether they were littermates with), with a corresponding +7.8% increase in puppies seen on their own between 2019 and 2020 (2019: 4.9% vs. 2020: 12.7%; *X*^2^ = 52.42, *p* < 0.001).

**Figure 6:**
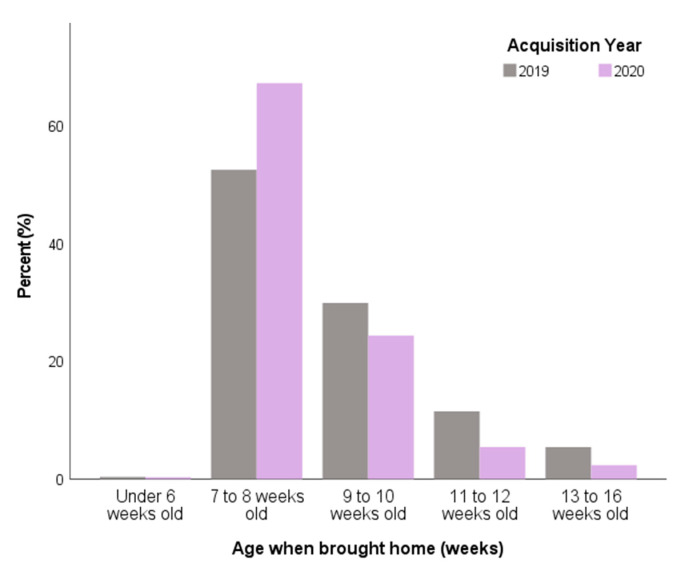
Age of puppies at acquisition (*n* = 5548) with comparison between 2019 owners (*n* = 1150) and Pandemic Puppy owners (*n* = 4398) in the UK.

Around one in seven puppies came from a breeder that had another litter for sale at the same time as the puppy that was purchased (of the same breed, 2019: 8.7% vs. 2020: 10.1%; of a different breed, 2019: 4.6% vs. 2020: 4.4%); however, this did not differ between Pandemic Puppies and 2019 puppies (*X*^2^ = 2.50, df = 4, *p* = 0.645). Pandemic Puppies were more likely to be purchased at a younger age, with two thirds (67.3%) purchased aged 7–8 weeks compared to 52.5% of 2019 Puppies (*X*^2^ = 113.01, *p* < 0.001) (Figure 6). A small minority of owners felt pressured by their puppy’s breeder to commit to purchasing their puppy (2019: 1.5% vs. 2020: 2.3%), which did not differ between 2019 puppies and Pandemic Puppies (*X*^2^ = 5.16, df = 3, *p* = 0.161).

**Figure 7:**
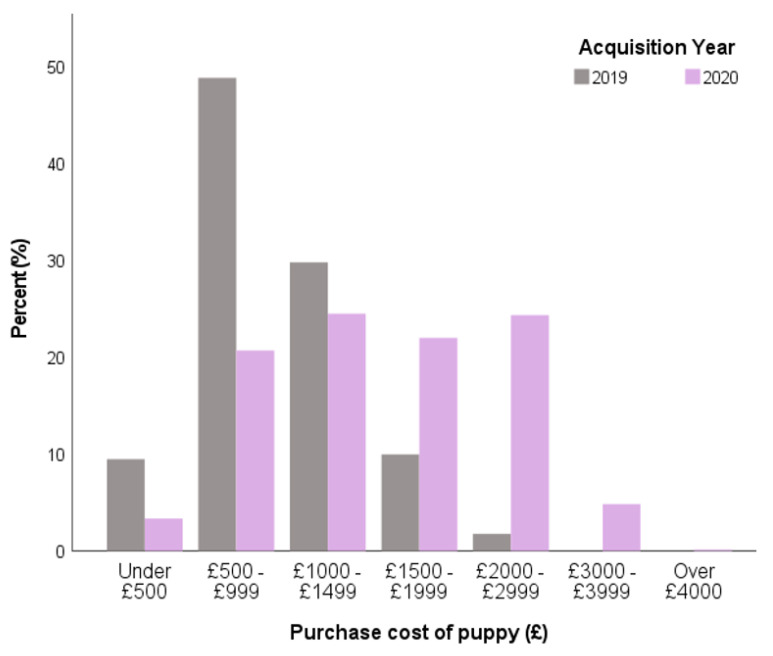
Purchase price of puppies, excluding any associated purchases such as food, collar, bowls, etc., with comparison between 2019 owners (*n* = 1022) and Pandemic Puppy owners (*n* = 3835) in the UK.

Owners of Pandemic Puppies paid significantly more for their puppy than 2019 owners, with one quarter of Pandemic Puppy owners (24.3%) paying £2000–2999 for their puppy compared to just 1.8% of 2019 puppy owners (*X*^2^ = 636.8, df = 6, *p* < 0.001, Figure 7).

Around two fifths (40.2%) of owners claimed to have heard of The Puppy Contract before purchasing their puppy; levels of awareness did not differ between 2019 puppy owners and Pandemic Puppy owners (2019: 41.5% vs. 2020: 40.2%; *X*^2^ = 0.53, df = 1, *p* = 0.466). Of those owners who were aware of The Puppy Contract (*n* = 1858), two thirds used the contract during their purchase, with levels of use the same across 2019 puppy and Pandemic Puppy purchasers (63.0% vs. 62.8%; *X*^2^ = 0.01, df = 1, *p* = 0.952). The most common reason for not using (either choosing not to use or being unable to use) The Puppy Contract was a belief that it was not needed as the owners were confident in their own purchasing decision, followed by the breeder not offering to use The Puppy Contract. Pandemic Puppy owners were significantly more likely to find their breeder did not agree to use The Puppy Contract when asked (Table 14).

#### Purchasing Behaviours: Multivariable Analysis

Multivariable logistic regression modelling identified seven variables related to purchase behaviour with significant association with Pandemic Puppies (Table 15). The Hosmer-Lemeshow test indicated acceptable model fit (*p* = 0.972).

### 3.8. Influence of COVID on Puppy-Purchasing Decisions in 2020

Of those households that purchased puppies during the 2020 Pandemic, 11.0% had not considered purchasing a puppy before the COVID-19 pandemic (*n* = 424), and a further 0.5% (*n* = 21) were unsure whether they had. More than two in five Pandemic Puppy purchasers felt their decision to purchase a puppy had been influenced by the COVID-19 Pandemic (41.5%, *n* = 1596), with a further 2.4% unsure as to whether it had influenced their decision (*n* = 106). Of those 2809 owners who felt the pandemic had influenced their decision, reasons are listed in Table 16, the most common of which was having more time to care for a dog (86.7%, *n* = 1378).

## 4. Discussion

This study characterises the nature of the Pandemic Puppy phenomenon in the UK and reveals some remarkable insights into how puppy purchasing in the UK has changed following the start of the first lockdown on 23 March 2020. These findings indicate that the surge in puppy purchasing reported during the 2020 phase of the pandemic was not simply pre-pandemic puppy purchasing at a greater scale, but that the COVID-19 pandemic was associated with several critical changes in why and how puppies were purchased, and by whom. Although many elements of the puppy purchasing process remained constant from 2019 to 2020, some of the pandemic-related changes documented in this study pose increased risks to the long-term welfare of puppies purchased during this period and are of practical importance. The pandemic prompted many households that had never previously considered owning a dog to purchase a puppy during this unprecedented period, with 2 in 5 Pandemic Puppy purchasers feeling that the pandemic had influenced their purchase in some way, predominantly because they had more time to care for a dog. Given that public health restrictions related to COVID-19 relaxed during July 2021, including a reduced emphasis on working from home as restrictions relax, and the Government furlough is scheme is due to end in September 2021, temporarily increased time to care for a dog acting as a major driver of puppy acquisition may result in increased relinquishment risk of this unique Pandemic Puppy population. Lack of time is a consistently documented risk factor for relinquishment of dogs [40,41,42,43,44,45,46], commonly in dogs 2 years of age or younger (70%) [41], and often precipitated by changes in the household [45]. It is possible that some societal changes seen during the pandemic in the UK, particularly around working from home, may continue permanently in some form for many employees [47], which may facilitate increased levels of dog ownership in the UK population. However, many roles including those in retail, manufacturing, leisure and hospitality will still require employees to travel to their place of work [48] and thus there are likely to be individual and regional differences in time-related relinquishments of puppies or dogs. Media reports suggest that increased relinquishment of Pandemic Puppies may have already begun [49,50,51,52,53]. Enhanced support mechanisms for Pandemic Puppy owners to avoid relinquishment of their dog (where this is in the dog’s best interests) are urgently needed, including implementation of training to help dogs to be left alone without distress, and awareness (and potentially increased provision) of services such as day care and dog walkers to avoid dogs being left alone for long periods of time, an existing problem for an estimated one in five of the UK dog population prior to the pandemic [54].

Mental health in the wider UK human population has deteriorated during the pandemic, with life satisfaction and happiness decreasing and anxiety levels increasing in adults [55]. Several findings from the current study indicate that Pandemic Puppies were often purchased in an attempt to mitigate some of these mental health challenges: Pandemic Puppy owners commonly sought out a dog to improve their own or their family’s mental health, significantly more than by 2019 puppy owners. When reporting on whether and why the pandemic influenced their puppy purchase, three of the top five reported reasons had a mental health component: to have ‘something happy’ to focus on, a reason to go outside to exercise more and a desire for more company due to being at home more. These findings are in agreement with many of the mental health benefits reported by existing dog owners during the pandemic, including making isolation easier to tolerate via offering companionship, having someone to talk to, physical connection and protection against loneliness, improving wellbeing via offering routine, purpose and motivation for life, exercise encouragement, and increased opportunity to socialise [34,56]. Some of these benefits were promoted to prospective puppy owners via media reports during the pandemic [57,58].

Although dogs play a variety of roles in 21st century society, restrictions on activity and social contact during the pandemic may have limited some of these functions. In support of this view, Pandemic Puppy purchasers were less likely to seek out puppies for specific non-working roles such as dog sports and conformational showing, which may have changed the demographics of the puppy population during 2020. Instead Pandemic Puppy owners increasingly selected their puppy’s breed/crossbreed based on anthropocentric lifestyle factors including being a size suited to the owners’ lifestyle, being easy to train and being good with children. more commonly than 2019 owners. International evidence suggests that shorter and smaller breeds are becoming more popular over time, supporting the concept that people attempt to purchase dogs that can fit their lifestyle niche [59]. The desired features of Pandemic Puppies could reflect the relative inexperience of this owner population. Puppy purchasing during the pandemic appears to have been driven partially by individuals and families seeking to purchase a dog for the first time, with two in five Pandemic Puppy owners having no previous dog ownership experience compared with one in three of the 2019 owners. The relative inexperience of Pandemic Puppy owners may pose risks to the future behaviour, welfare and homing status of this puppy population. First-time dog ownership is associated with an increased risk of relinquishment [60,61], and it has been argued that owners’ knowledge and attitudes, including ignorance of species-specific behaviours, unrealistic expectations for the roles pets play in children’s lives, and the expense and time required for dog ownership and caretaking contribute to this high relinquishment [61]. First-time dog ownership has been associated with increased perceived ‘costs’ (or burden) of dog ownership [62], which may reflect a lack of understanding of the day-to-day responsibility of dog ownership prior to acquisition. In addition, first-time dog owners are more likely to report problem behaviours in their dog, including aggression [63], separation-related problems [63], noise phobias [63,64,65], nervousness [66] and overexcitement [63,66]. Whether these perceived problems represent true behavioural pathologies or are within the realm of ‘normal’, non-pathological dog behaviour, with new owners more inclined to consider them undesirable due to their inexperience, has been speculated as a reason for this association [63,66]. Previous data indicate that greater animal care knowledge is associated with an increased expectation for effort required in owning a dog [67], and those with arguably the most experience of dogs—professionals from the canine and/or animal care sector—were significantly less likely to be Pandemic Puppy owners. This may also reflect concerns over the Pandemic Puppy phenomenon from informed parties, who may have greater awareness of welfare risks to puppies and breeding dogs during this period of high demand. Indeed, similar avoidance of other companion animal acquisition phenomena that put animal welfare at greater risk have been reported in this professional group, for example, a reduced preference towards brachycephalic cats and rabbits [68,69]. Given the relatively inexperienced demographic of Pandemic Puppy owners, and the consequent potential negative outcomes for dog and owner, provision of increased support and education for Pandemic Puppy owners is likely to be required by canine behaviour and welfare professionals and organisations to maintain the welfare of this vulnerable puppy population.

Owners of Pandemic Puppies were more likely to live in households with children. Despite this, only 4.1% of this group explicitly stated that they purchased a puppy to keep their children busy during the pandemic. However, compared with 2019 owners, Pandemic Puppy owners were more likely to want to own a dog to improve their/their family’s mental health. In addition, Pandemic Puppy owners were more likely to desire a dog breed they perceived to be good with children compared with 2019 owners. Although dogs may have offered positive benefits to families during lockdown [56], public health measures to limit the spread of COVID in the UK were associated with an increase in paediatric dog bite emergency department attendances, potentially due to children spending more time at home, with greater exposure to dogs [70]. Bites peaked during May to July 2020, with the authors of that study speculating that increased exposure to dogs due to the acquisition of Pandemic Puppies as 2020 progressed may have been one reason for this increase [70]. The desire for a dog that is safe with children has been cited in several studies characterising the ‘ideal companion dog’ [71,72] and perceived suitability for living with children has driven the recent surge in brachycephalic dogs [73]. However, with no robust evidence that breed is a risk or protective factor for dog bites [74], urgent owner education is needed to raise awareness of the dog bite risk to children and promote safe interactions with dogs within and outside of the household.

Although one in ten Pandemic Puppy owners had not considered owning a dog before the pandemic, the proportion of owners who reported that they carried out no pre-purchase research did not differ between 2019 and 2020, and was consistently low (3.0%). Significantly more Pandemic Puppy owners performed pre-purchase research than 2019 owners, albeit this difference can be explained by a greater number of 2019 owners feeling sufficiently experienced as dog owners not to undergo this process. Commonly used pre-purchase information sources both before and during the pandemic were breed/crossbreed-specific online resources, The Kennel Club website and talking to dog breeders. Pandemic Puppy owners were more likely to conduct pre-purchase research on animal charity websites (+16.2%) or by talking to friends and family who had owned a dog (+10.5%), highlighting the importance of reliable online resources but also the power of anecdote over evidence, with peer-peer and family communication more common, which although trusted may be of variable quality. With social distancing in place throughout the pandemic, owners were less likely to talk to strangers that owned dogs of the breed they eventually purchased, albeit this may also be an unreliable source of information. Although The Kennel Club reported that searches using its “Find a Puppy” online tool increased during the pandemic compared to the same period in 2019 [3,4], Pandemic Puppy owners were significantly less likely to find their puppy using this tool, which may reflect exhaustion of the section of the market that advertised via this website.

Both before and during the pandemic, puppy owners valued dog breeders who would allow them to see their puppies’ mother (dam), they felt cared for their dogs, and whom they felt were trustworthy and offered good communication, with Pandemic Puppy owners more likely to seek out breeders who offered good communication. The increased desire for a communicative breeder in 2020 may reflect owners becoming increasingly cautious of unscrupulous breeders cashing in on demand, given media reports and public campaigns ran by DEFRA (‘Petfishing’ [75]), Dogs Trust (‘DogFishing’ [5]) and The Kennel Club (#BePuppywise [76]) to educate prospective owners on this issue. Despite seeking a breeder who was perceived to care for their dogs, Pandemic Puppy owners were less likely to seek out a breeder who was a member of the Kennel Club Assured Breeders Scheme (ABS) or performed health tests for the breed/crossbreed they wanted. It is possible that prospective owners were not aware of these schemes prior to their purchase, or that scarcity of ABS breeders and/or those advertising health testing during the pandemic influenced prospective owners’ priorities when looking for a breeder. Understanding how owners define a ‘caring breeder’ is an important future research area given that this was an important characteristic for owners in both years, yet many puppies were purchased from breeders who were not following recommended or even legal standards.

The common desire by prospective owners for a breeder who allowed prospective owners to see their puppy’s mother (dam) may reflect the success of the campaign that led to Lucy’s Law being passed into English legislation in 2019, which made it illegal to sell a puppy without showing its mother in the place it was born [77]. Despite this desire, this outcome was not fully achieved in many purchases during both 2019 or 2020, with one in seven puppies seen without their mother (dam) in 2019 and 1 in 4 in 2020. Understanding why owners make compromises during the purchasing process requires further research; however, previous data has demonstrated that actions differing from plans when acquiring a dog are common, particularly regarding the source of the dog [78,79]. It is possible that the ‘sellers’ market’ created in 2020, where demand outstripped supply, may have pushed some owners to make suboptimal decisions in their desperation to purchase a puppy. In May 2020, the media reported a UK ‘puppy shortage’ [80]. Scarcity messaging of this type during the pandemic led to panic buying of many household products—most infamously toilet rolls—with such messaging potentially heightening the urgency consumers feel to buy impulsively [10]. It is possible these messages had a similar effect on puppy purchasing; owners of Pandemic Puppies were willing to pay significantly more for their puppy than 2019 owners, with one in four owners paying over £2000 for their puppy, which was previously extremely rare with fewer than one in fifty owners paying this price in 2019. This was complemented by Pandemic Puppy owners being more likely to place a deposit on their puppy before they had seen them, leaving owners open to ‘PetFishing’ [75] and potentially limiting their freedom to change their minds if unhappy with the puppy or its environment when they met in person. Taken together with population statistics, it is likely that the unprecedented scale of owners strongly driven to purchase a puppy in 2020 enhanced the market for unscrupulous breeding of puppies, and, indeed, illegal imports to meet this demand, as reported by Dogs Trust [81].

Public health measures to control the pandemic introduced restrictions that altered the pre-purchase and purchase behaviour of puppy purchasers. Pandemic Puppy owners were less likely to visit their breeder in person to view their puppy, reducing from four in five during 2019 to three in five owners during 2020 physically seeing their puppy prior to the day of purchase. Instead, during the pandemic, prospective owners were significantly more likely to view their puppy via live video calls or pre-recorded videos or photos. For those who did visit in person prior to bringing their puppy home, the number of visits was significantly lower. Limits to contact and viewing of the breeder’s property continued during the purchase, with Pandemic Puppy owners less likely to collect their puppy from inside the breeders’ property, replaced by doorstep/garden collections or deliveries to the new owner. Although these behaviours were unavoidable if adhering to ‘stay at home’ orders during some periods of 2020, these changes to the pre-purchase process increase the likelihood of puppy purchasers purchasing from breeders or dealers who were trying to conceal the poor environment and conditions their puppies were raised in, or indeed hide that the puppies they were selling weren’t raised at that location at all [75]. During the purchase visit, Pandemic Puppy owners were less likely to see their puppy with its littermates compared to 2019 owners. This may further indicate an increased number of unscrupulous puppy dealers in 2020 compared to 2019, who are reported to often separate puppies from their litter [75]. Increased educational efforts to re-emphasise the importance of seeing a puppy in person prior to purchasing them, collecting them from within the breeder’s property where they are able to see and interact with the puppies’ mother and littermates is needed to promote canine welfare.

Health testing of breeding dogs, both phenotypic screening (e.g., hips, elbows, knees, eyes, respiratory testing) and DNA (genetic) tests are a critical tool in improving the health and welfare of future generations of dogs, including both pedigree and intentional ‘designer’ crossbreeds. Fewer Pandemic Puppy owners asked to see screening test results during the purchase process, a concerning trend previously documented in the purchase of brachycephalic breeds [15]. The lack of requests to see health records may reduce the priority placed on health by breeders, by reducing demand for healthy, tested dogs. While market forces of supply and demand are not being effectively applied to canine health, breed health is unlikely to improve substantially unless a sufficient proportion of breeders are intrinsically motivated to carry out health testing themselves.

## 5. Conclusions

The COVID-19 pandemic impacted all stages of puppy purchasing in the UK, from the reasons why prospective owners wanted to purchase a puppy, through to the actual puppy purchase. Given the nature of some of these changes, including an increase in inexperienced owners purchasing a puppy for the first-time, fewer puppies being seen in the environment where they were raised, and a reduced emphasis on canine health, combined with the reportedly large scale of purchasing during this period of 2020, this unique Pandemic Puppy population may face significant welfare problems in the future. In the short-term, whether owners are able to continue owning these now adolescent to young adult dogs as COVID-19 restrictions change in the UK is a major concern, given the initial motivation to purchase a dog during the pandemic being primarily based on the increased time this unique period offered. In the medium-long term, threats to the health and behaviour of this population include the potentially unsuitable environments many of these puppies might have been bred and raised in negatively impacting their behaviour, and the low regard for genetic health during many purchases potentially increasing the risk of breed-related disorders. It is clear that greater support from animal welfare organisations, veterinary professionals and animal behavioural professionals will be required to avoid, where possible, and address, where present, negative welfare outcomes likely to arise in this population. This study has further highlighted many of the deficiencies in UK puppy-purchasing culture both before and during the pandemic, and the need for ongoing education of prospective owners, as well as legislation to improve breeding practices, where consumer demand is not appropriately directed to effect positive welfare changes.

## Figures and Tables

**Table 1:** Final multivariable model for owner demographic and lifestyle variables associated with purchasing a ‘Pandemic Puppy’ in the UK during 2020. * Confidence interval.

Variable	Category	Odds Ratio	95% CI *	*p*-Value
Children in Household	Yes	1.27	1.10 to 1.49	0.002
	No	Reference		
Previous dog ownership	Yes	0.80	0.69 to 0.93	0.004
	No	Reference		
Furloughed	Yes	0.77	0.66 to 0.89	0.001
	No	Reference		
Employed in veterinary/animal care sector	Yes	0.55	0.46 to 0.74	<0.001
	No	Reference		

**Table 2:** Comparison of reasons why UK owners wanted to purchase a dog, compared between 2019 puppy (*n* = 1148) and Pandemic Puppy (*n* = 4360) owners in the UK.

Reason (*n* = 5508)	Acquisition Year		Statistics	
	2019 (%) (*n* = 1148)	2020 (%) (*n* = 4360)	*X* ^2^	*p*-Value
Companionship for myself	64.5	64.7	0.01	0.911
To encourage myself/my family to walk and exercise	47.6	53.6	12.91	<0.001
To improve my/my family’s mental health	38.1	46.9	28.52	<0.001
Companionship for other adult(s) in my household	34.8	37.0	1.91	0.167
Due to the loss of a previous dog in my household	30.8	28.6	2.30	0.130
Companionship for my children	19.1	24.0	12.51	<0.001
To keep me/my family busy	22.8	23.7	0.40	0.526
Companionship for my other dog(s)	25.7	19.9	18.28	<0.001
As a working dog for a specific role (e.g., gundog, security, sniffer/tracking, herding, medical detection, assistance/therapy dog)	10.4	7.9	7.40	0.007
For a specific non-working role (e.g., dog sports, showing, etc.)	8.1	4.1	30.44	<0.001
Due to the ageing of another dog/other pet in my household	0.9	0.7	0.42	0.516
For breeding	1.1	0.6	3.71	0.054
Due to the death of another (non-dog) pet in my household	0.2	0.4	1.64	0.201
Companionship for my other (non-dog) pets	0.0	0.0	0.527	0.468

**Table 3:** Characteristics sought by owners when selecting a particular breed/crossbreed puppy to purchase, with comparisons between 2019 puppy (*n* = 1123) and Pandemic Puppy owners (*n* = 4281) in the UK.

Characteristic (*n* = 5404)	Acquisition Year		Statistics	
	2019 (%) (*n* = 1123)	2020 (%) (*n* = 4281)	*X* ^2^	*p*-Value
Good companion	67.9	70.8	3.63	0.057
Size suited to my lifestyle	59.1	64.9	13.00	<0.001
Generally healthy breed/crossbreed	45.3	48.4	3.42	0.064
Good with children	38.7	47.9	29.84	<0.001
Easy to train	34.7	43.3	27.11	<0.001
Appearance/looks	39.3	39.7	0.08	0.777
I’ve owned this breed or crossbreed before	40.1	33.0	19.66	<0.001
Exercise encouragement	29.9	33.0	3.76	0.053
Friends or family currently own this breed/crossbreed	19.8	24.0	8.83	0.003
I grew up with or had childhood experiences with this breed/crossbreed	18.5	18.8	0.03	0.857
Hypoallergenic	15.2	18.0	4.64	0.031
Low grooming needs	14.2	16.4	3.47	0.063
Long life expectancy	15.9	14.2	1.94	0.163
Working ability of the breed/crossbreed	17.5	13.2	14.19	<0.001
Affordable purchase cost of puppies	8.4	9.3	0.88	0.349
Affordable cost of upkeep	7.7	8.0	0.15	0.696
Low exercise requirements	5.0	5.5	0.52	0.469
Popularity of the breed/crossbreed	4.9	4.4	0.583	0.445
Other perceived temperament/personality traits	3.8	3.2	0.93	0.336
None of these options—I did not have any specific characteristics I was looking for	3.2	1.6	12.33	<0.001
I’ve always wanted to own this breed/crossbreed	0.5	0.5	0.08	0.772
Low or non-shedding	0.6	0.4	1.03	0.310
Specific genetic characteristics of the breed/crossbreed	0.5	0.4	0.40	0.530
Celebrity/Influencer endorsement/ownership	0.4	0.1	3.07	0.080
None of these options—someone else in the household selected the breed/crossbreed of our puppy	0.3	0.1	2.08	0.150

**Table 4:** Characteristics of breeders that prospective owners sought out, with comparisons between 2019 puppy (*n* = 1101) and Pandemic Puppy owners (*n* = 4178) in the UK.

Breeder Characteristic (*n* = 5279)	Acquisition Year		Statistics	
	2019 (%) (*n* = 1101)	2020 (%) (*n* = 4178)	*X* ^2^	*p*-Value
They would allow me to see the puppies’ mother (dam)	90.5	88.9	2.29	0.130
Someone I felt cared for their dogs	81.7	84.6	5.76	0.016
Someone I felt was trustworthy	79.2	78.9	0.03	0.860
Good communication with me	74.2	78.8	10.62	0.001
They performed health tests for the breed/crossbreed I wanted	66.9	62.1	8.90	0.003
They would allow me to see the puppies’ father (sire)	44.2	42.1	1.69	0.193
Availability of the breed I wanted	39.2	41.8	2.43	0.119
Lived within the distance I was willing to travel	37.5	40.5	3.34	0.067
Availability of puppies at the time I wanted	38.3	40.1	1.13	0.288
Reasonably priced puppies	35.5	36.3	0.25	0.614
Bred the colour of the breed/crossbreed I wanted to purchase	21.0	21.8	0.31	0.578
A member of the Kennel Club Assured Breeders Scheme	25.2	20.2	12.54	0.001
The dogs they bred from had been awarded prizes at dog shows	11.3	8.2	10.25	0.001
Someone I already knew	1.1	1.1	0.011	0.916
Registered with the local Council	0.5	0.6	0.144	0.704
They bred dogs with specific working and/or sporting characteristics	1.0	0.6	2.74	0.098
They were a member of a specific breed/crossbreed club or association	0.2	0.3	0.24	0.627
They registered their puppies with an international canine registration body (e.g., FCI)	0.0	0.1	0.79	0.374

**Table 5:** Final multivariable model for pre-purchase motivations associated with purchasing a ‘Pandemic Puppy’ during 2020 compared with a 2019 puppy in the UK. * Confidence interval.

Question	Variable	Category	Odds Ratio	95% CI *	*p*-Value
Motivation for a dog	Companionship for other adult(s) in my household	Yes	0.83	0.69 to 0.98	0.036
		No	Reference		
	To improve my/my family’s mental health	Yes	1.22	1.04 to 1.43	0.014
		No	Reference		
	Specific non-working role	Yes	0.69	0.52 to 0.93	0.014
		No	Reference		
Household driver for dog	Responding owner alone the main driver of puppy purchase	Yes	0.82	0.70 to 0.95	0.008
		No	Reference		
Motivation for breed	Friends/family own	Yes	1.22	1.02 to 1.47	0.034
		No	Reference		
	Good with children	Yes	1.33	1.14 to 1.56	0.001
		No	Reference		
	Size suits lifestyle	Yes	1.17	1.01 to 1.36	0.049
		No	Reference		
	Easy to train	Yes	1.24	1.06 to 1.45	0.008
		No	Reference		
Desirable characteristics of breeder	Good communication	Yes	1.34	1.12 to 1.60	0.001
		No	Reference		
	Did health tests	Yes	0.76	0.64 to 0.89	0.001
		No	Reference		
	Member of KC ABS	Yes	0.75	0.63 to 0.89	0.001
		No	Reference		

**Table 6:** Sources of information used when researching dog ownership and/or which breed/crossbreed to purchase prior to purchasing a puppy, with comparisons between 2019 puppy (*n* = 591) and Pandemic Puppy owners (*n* = 2771) in the UK.

Information Source (*n* = 3362)	Acquisition Year		Statistics	
	2019 (%) (*n* = 591)	2020 (%) (*n* = 2771)	*X* ^2^	*p*-Value
Talking to friends or family who own or had owned a dog	54.8	65.3	23.15	<0.001
A breed/crossbreed-specific online resource (e.g., website/forum)	54.8	60.1	5.51	0.019
The Kennel Club website	50.6	54.2	2.61	0.106
Talking to a dog breeder	53.6	49.7	2.98	0.084
Social media sites, e.g., Facebook, Instagram	43.5	47.2	2.65	0.103
An animal charity website, e.g., Dogs Trust, RSPCA, PDSA, etc.	28.8	45.0	52.89	<0.001
Book(s)	30.1	35.0	5.25	0.022
My veterinary professional (e.g., veterinary surgeon, veterinary nurse)	14.0	11.3	3.44	0.064
Dog-specific magazine(s)	6.4	5.4	1.03	0.311
Other digital sources (e.g., articles on the internet, TV shows)	3.7	3.2	0.40	0.528
Talking to a non-veterinary animal professional (e.g., dog trainer, behaviourist)	1.0	1.0	0.01	0.946
Talking to current dog owners that I met (that were not friends or family)	2.4	1.0	7.29	0.007
I can’t remember	0.8	0.3	3.19	0.074
From professional experience with dogs in my workplace	0.7	0.3	1.57	0.211
Seeking practical experience of caring for dogs (e.g., dog sitting, fostering)	0.2	0.1	0.02	0.887

**Table 7:** Places owners found their puppy with comparisons between 2019 puppy (*n* = 1100) and Pandemic Puppy owners (*n* = 4177) in the UK.

Method of Initially Finding Puppy (*n* = 5277)	Acquisition Year		Statistics	
	2019 (%) (*n* = 1100)	2020 (%) (*n* = 4177)	*X* ^2^	*p*-Value
An animal specific selling website, e.g., Pets4Homes, Champdogs	34.5	45.0	32.64	<0.001
I already knew the breeder (e.g., colleague, friends, family, repeat purchase)	26.8	19.8	25.82	<0.001
Recommendation from a friend	11.5	14.3	5.73	0.017
The Kennel Club website ‘Find A Puppy’ search	16.9	11.8	20.01	<0.001
A general selling website, e.g., FreeAds, Gumtree, Preloved	9.3	9.3	0.01	0.948
A social media breed/crossbreed specific group	8.5	8.4	0.01	0.977
The breeder’s website	8.7	7.6	1.43	0.232
The breeder’s social media account	6.5	6.0	0.52	0.471
Recommendation from another breeder/stud dog owner	1.2	2.0	3.32	0.069
Recommendation from someone who is not a colleague, friend, family member or animal professional	1.9	1.6	0.68	0.411
Dog specific magazine(s)/newspaper(s)	0.3	0.6	1.37	0.242
An advert seen in another online location	0.6	0.3	3.56	0.059
The breeder contacted me directly following general expression of interest for a puppy	0.1	0.3	1.14	0.285
Local newspaper advert	0.0	0.2	2.37	0.123
Recommendation from an animal professional (e.g., veterinary surgeon, veterinary nurse, dog trainer)	0.2	0.2	0.01	0.919
Recommendation from a stranger	0.2	0.2	0.01	0.947
An advert in a local shop	0.1	0.0	1.03	0.310
A physical advert in another location (e.g., vets noticeboard)	0.2	0.0	7.60	0.006

**Table 8:** Puppy viewing practices prior to the date the puppy was brought home (*n* = 5280) with comparison between 2019 owners (*n* = 1100) and Pandemic Puppy owners (*n* = 4180) in the UK.

Viewing Practice (*n* = 5280)	Acquisition Year		Statistics	
	2019 (%) (*n* = 1100)	2020 (%) (*n* = 4180)	*X* ^2^	*p*-Value
Yes—visited the breeder’s property in person	80.6	59.6	164.63	<0.001
Yes—saw photos or a pre-recorded video of my/our puppy	31.2	51.6	142.23	<0.001
Yes—saw my/our puppy on a live video call with their breeder	6.5	28.9	235.18	<0.001
No—I did not ask to see my/our puppy	4.7	4.9	0.04	0.842
No—the purchase was a rapid decision, so my puppy was brought home on the same day they were viewed	5.1	2.7	16.45	<0.001
No—I was unable to visit due to the breeder being too far away to travel to	1.7	2.0	0.60	0.437
No—I wanted to see my/our puppy, but the breeder refused	0.1	1.1	9.73	0.002
No—but a friend/relative visited the breeder’s property on my behalf	0.1	0.2	0.70	0.402
Yes—I saw my puppy in person but not at the breeder’s property	0.0	0.1	1.05	0.306
No—I only spoke to the breeder by telephone	0.1	0.1	0.04	0.833
No—I asked to visit but personal circumstances meant I was unable to visit myself or get someone else to visit on my behalf	0.1	0.0	0.29	0.590

**Table 9:** Acquisition levels of first-choice breeds by owners and reasons for not purchasing their first-choice breed with comparison between 2019 puppy owners (*n* = 1118) and Pandemic Puppy owners (*n* = 4256) in the UK.

First Choice Breed? (*n* = 5374)	Year	
	2019 (%) (*n* = 1118)	2020 (%) (*n* = 4256)
Yes	91.6	86.6
No, I could not find a seller that had puppies available at the time for my first choice	1.2	3.8
No, first choice too expensive	0.8	2.8
No, I/we didn’t have a specific choice in mind	3.0	2.7
No, I could not find a breeder I felt happy buying my puppy from for my first choice	0.4	1.3
No, I/we changed our mind	1.0	0.8
No, I/we wanted a rescue dog but were unable to source one	0.4	0.7
No, but I had several breeds (≤3) I was interested in and got one of them	0.4	0.3
No, puppies of my first choice were too far away	0.3	0.2
No, our planned purchase of our first choice fell through	0.3	0.2
No, but I had several breeds (>3) that I was interested in and got one of those	0.6	0.5

**Table 10:** Final multivariable model for pre-purchase behaviours associated with purchasing a ‘Pandemic Puppy’ in the UK during 2020. * Confidence interval.

Question	Variable	Category	Odds Ratio	95% CI *	*p*-Value
Pre-purchase research	Research conducted	Yes	1.49	1.01 to 2.20	0.044
		No	Reference		
Sources of pre-purchase information	Charity website	Yes	1.98	1.56 to 2.51	<0.001
		No	Reference		
	Family/friends	Yes	1.51	1.18 to 1.93	0.001
		No	Reference		
	Owners of breed—strangers	Yes	0.36	0.17 to 0.79	0.011
		No	Reference		
Find breeder	KC website	Yes	0.56	0.43 to 0.74	<0.001
		No	Reference		
Viewing puppy prior to purchase	Visited in person	Yes	0.24	0.11 to 0.53	<0.001
		No	Reference		
	Live video calls	Yes	3.42	2.21 to 5.30	<0.001
		No	Reference		
	Video and photo recordings	Yes	1.46	1.15 to 1.85	0.002
		No	Reference		
Number of visits to breeder (*n*)			0.89	0.83 to 0.96	0.001

**Table 11:** Locations owners received their puppy with comparison between 2019 puppy owners (*n* = 1100) and Pandemic Puppy owners (*n* = 4180) in the UK.

Location (*n* = 5280)	Acquisition Year		Statistics	
	2019 (*n* = 1100)	2020 (*n* = 4180)	*X* ^2^	*p*-Value
The breeder’s property—from inside their home	84.7	51.0	407.7	<0.001
The breeder’s property—from outside their home, e.g., doorstep, garden	5.5	29.8	277.0	<0.001
The breeder’s property—an outdoor kennels, barn or outbuilding	13.2	14.8	1.92	0.166
The breeder delivered my puppy to my property	1.0	5.2	36.76	<0.001
A service station	0.0	1.4	15.97	<0.001
A car park	0.3	1.1	6.490	0.011
A courier/pet transporter delivered my puppy to my property	0.5	0.9	1.87	0.172
Breeder/courier met us half way (location unspecified)	0.1	0.5	3.30	0.069
The breeder’s workplace or non-residential property (e.g., holiday home)	0.3	0.4	0.41	0.520
A veterinary practice	0.1	0.2	0.71	0.398
Other transport location (e.g., train station or ferry port)	0.0	0.2	1.85	0.174
Other public location (e.g., park, shop, hotel)	0.0	0.2	2.37	0.123
An airport	0.1	0.1	0.06	0.801
A lay-by	0.0	0.1	0.790	0.374
A friend collected and delivered my puppy for me	0.1	0.0	0.28	0.594

**Table 12:** Owners’ requests for information related to health testing of their puppies’ parents with comparison between 2019 puppy owners (*n* = 908) and Pandemic Puppy owners (*n* = 3509) in the UK.

Test Type	Request and Provision of Information	Acquisition Year	
		2019 (%) (*n* = 908)	2020 (%) (*n* = 3509)
Results of DNA (genetic) tests (*n* = 4296)	Yes, and they provided me with it	48.5	40.7
	Yes, but they could not provide me with it	2.6	4.3
	No, I did not ask about this	41.0	46.7
	No, I do not believe there are any tests available for my puppy’s breed/crossbreed	8.0	8.2
Results of veterinary screening tests (e.g., hips, elbows, knees, eyes, respiratory testing) (*n* = 4394)	Yes, and they provided me with it	58.1	46.4
	Yes, but they could not provide me with it	3.7	4.8
	No, I did not ask about this	30.8	41.2
	No, I do not believe there are any tests available for my puppy’s breed/crossbreed	7.4	7.6

**Table 13:** Other dogs that were seen at the seller’s premises on the day of purchase of the puppy with comparison between 2019 puppy owners (*n* = 1073) and Pandemic Puppy owners (*n* = 4095) in the UK.

Other Dogs and Their Relationship to the Purchased Puppy (*n* = 5168)	Acquisition Year		Statistics	
	2019 (*n* = 1073)	2020 (*n* = 4095)	*X* ^2^	*p*-Value
Their mother (dam)	85.7	75.1	54.74	<0.001
Their littermates	84.9	72.1	74.21	<0.001
Another dog(s) they were not related to (e.g., another breed)	33.2	25.7	23.88	<0.001
Their father (sire)	26.9	23.5	5.41	0.020
I only saw my/our puppy	4.9	12.7	52.42	<0.001
Adult dog(s) they were related to (e.g., aunts, grandparents, older siblings)	8.6	5.5	13.74	<0.001
Other puppies (unsure if they were littermates)	4.3	2.8	6.40	0.011
I’m not sure, I wasn’t the person who collected my/our puppy	0.2	0.9	5.84	0.016
Other puppies my puppy was related to (but not littermates)	0.4	0.3	0.32	0.572
I don’t remember	0.2	0.2	0.01	0.953
Another adult dog(s) the breeder claimed were my puppy’s parent(s), but I’m not sure	0.3	0.1	1.36	0.243
Other puppies they were not related to (e.g., another breed)	0.0	0.0	0.26	0.609

**Table 14:** Reasons owners chose not to or were unable to use The Puppy Contract during their puppy purchase with comparison between 2019 puppy owners (*n* = 151) and Pandemic Puppy owners (*n* = 538) in the UK.

Reason (*n* = 689)	Acquisition Year		Statistics	
	2019 (%) (*n* = 151)	2020 (%) (*n* = 538)	*X* ^2^	*p* Value
I didn’t feel it was needed for the sale of my puppy as I was confident in my own purchasing decision	21.9	28.2	0.42	0.120
The breeder did not offer to use The Puppy Contract	20.5	22.4	0.25	0.615
I used a written contract with the breeder, but not The Puppy Contract	15.9	12.4	1.24	0.266
I didn’t feel it was needed, as the breeder is a friend, family member or someone I’ve bought a puppy from before	13.9	10.2	1.65	0.199
I didn’t know enough or feel confident enough about The Puppy Contract to use it	7.2	6.3	0.19	0.667
I forgot to use it when purchasing my puppy	2.6	6.1	2.80	0.94
I didn’t think it was relevant and/or possible for the sale of my puppy	7.3	5.6	0.62	0.430
The breeder didn’t agree to use The Puppy Contract when asked	0.7	3.9	4.0	0.046
I used a verbal contract with the breeder, but not The Puppy Contract:	2.6	2.2	0.09	0.760
I felt uncomfortable proposing its use and/or feared repercussions of suggesting it	0.7	2.0	1.31	0.251
I have negative views on the value of The Puppy Contract	2.0	0.9	1.16	0.283

**Table 15:** Final multivariable model for purchasing behaviours associated with purchasing a ‘Pandemic Puppy’ in the UK during 2020. * Confidence interval.

Question	Variable	Category	Odds Ratio	95% CI *	*p*-Value
Pre-purchase deposit	Deposit	No—I wasn’t asked	0.79	0.57 to 1.08	0.785
		No—there was no time	0.49	0.20 to 1.20	0.117
		Yes—after I saw my puppy	0.70	0.61 to 0.94	0.020
		Yes—before I saw my puppy	Reference		
Cost of puppy	Purchase price	Under £500	0.01	0.01 to 0.02	<0.001
		£500–999	0.02	0.01 to 0.03	<0.001
		£1000–1499	0.04	0.02 to 0.06	<0.001
		£1499–1999	0.12	0.06 to 0.21	<0.001
		>£2000	Reference		
Age when brought home	Age	Under 6 weeks	1.98	0.36 to 11.02	0.432
		7 to 8 weeks	3.30	2.03 to 5.37	<0.001
		9 to 10 weeks	1.95	1.18 to 3.21	0.009
		11 to 12 weeks	0.89	0.51 to 1.57	0.089
		13 to 16 weeks	Reference		
Health tests	Screening tests	Yes, and they provided me with it	0.37	0.30 to 0.45	<0.001
		Yes, but they couldn’t provide it	0.43	0.26 to 0.70	<0.001
		No, I do not believe there are any tests available	0.96	0.67 to 1.37	0.830
		No, I did not ask about this	Reference		
Collect puppy	Inside at breeders	Yes	0.30	0.23 to 0.39	<0.001
		No	Reference		
	Garden at breeders	Yes	4.73	3.20 to 7.00	<0.001
		No	Reference		
	Breeder delivered	Yes	6.85	2.68 to 17.51	<0.001
		No	Reference		
Who puppy was with	Saw littermates	Yes	0.72	0.56 to 0.93	0.011
		No	Reference		

**Table 16:** Reasons why the COVID-19 pandemic influenced decisions to purchase a puppy (*n* = 2809) in the UK during 2020.

Reason (*n* = 2809)	%	*n*
I/we had more time to care for a dog	86.7	1378
I/we wanted something happy to focus on	30.0	477
I/we wanted a reason to go outside to exercise more	29.6	470
I/we wanted more company due to being at home more	24.5	390
I/we had extra money to spend that I/we would have usually spent on other things	17.7	282
I/we wanted more company as family and/or friends were unable to visit me/us	9.1	144
I/we were bored due to the restrictions imposed by lockdown	4.3	69
My child/children were at home and I/we wanted something to keep them busy	4.1	65
I/we experienced mental health challenges due to the pandemic, that I/we wanted a puppy to help us with	0.9	15
I/we wanted a dog from another source (e.g., rescue) but were unable to due to pandemic, so bought a puppy instead	0.8	13
My/our existing dog experienced mental health challenges due to the pandemic, that I/we wanted a puppy to help them with	0.1	2

## Data Availability

The data presented in this study are available on request from the corresponding author.

## Supplementary Materials

The following are available online at https://www.mdpi.com/article/10.3390/ani11092500/s1, File S1: Questionnaire, File S2: Qualitative Coding Framework.
