# Supplementary material for: Pandemic Puppies: Characterising Motivations and Behaviours of UK Owners Who Purchased Puppies during the 2020 COVID-19 Pandemic
Source: Animals (Basel). 2021 Aug 25;11(9):2500. doi: 10.3390/ani11092500 (PMC8468924; doi:10.3390/ani11092500)
Supplement: Supplementary file 1 [file animals-11-02500-s001.zip › animals-1332546-supplementary.pdf]

<u>CONSENT QUESTIONS AND INCLUSION CRITERIA:</u>

Q1
<u>I confirm that:</u>
<u>[SURVEY LOGIC – If no to any question take to disqualification page]</u>

1. I am over 18 years of age
2. I am a resident of the UK
3. I have read and understood the above information and give consent for my answers to be used for this research study and any resulting publications
4. I brought a puppy home <u>aged under 16 weeks</u> during 2019 OR 2020

<u>ESSENTIAL QUESTIONS (ALL OWNERS):</u>

Q2
<u>When did you bring your puppy home?</u>
Drop down month list
Drop down year list (2019 & 2020)

Q3
<u>Please select the breed or crossbreed of dog you are answering for.</u>
Drop down list
Not on the list (please specify) **[free text]**

Q4
<u>Is your puppy/dog registered with The Kennel Club?</u>
Yes
Not applicable – crossbreed
Not applicable – breed not registered with The Kennel Club
I'm not sure
No
No – registered with another canine registration body, e.g. a working dog registry (please specify) **[free text]**

Q5
<u>What is your puppy's/dog's date of birth? Please enter in the format DD/MM/YY</u>
N.B. If you are unsure, please leave relevant part as '00'
Date text field

Q6
<u>What sex is your puppy/dog?</u>
Male
Female

Q7
<u>Is your puppy/dog insured?</u>
Yes
No – and I do not plan to insure them
No – but I plan to insure them in the future
No – they were insured but I have since cancelled or did not renew their policy
No – I have never heard of pet insurance
No – other **[free text]**

Q8
<u>What were the main reasons your household wanted to acquire a dog?</u>
<u>Please select **all options** that apply</u> **[RANDOMISE LIST FOR EACH SURVEY TO AVOID BIAS]**
Companionship for myself

Companionship for my children
Companionship for other adult(s) in my household
Companionship for my other dog(s)
To keep me/my family busy
To encourage myself/my family to walk and exercise
To improve my/my family's mental health
Due to the loss of a previous dog in my household
As a working dog for a specific role (e.g. gundog, security, sniffer/tracking, herding, medical detection, assistance/therapy dog)
Other reason not listed above **[free text]**

Q9
Did you or your household carry out any research into owning dog and/or which breed/crossbreed to buy before you purchased your puppy?

1. No **[SURVEY LOGIC – skip question 10]**
2. No – but I am already an experienced dog owner **[SURVEY LOGIC – skip question 10]**
3. Yes – please describe in your own words the research you carried out **[free text]**

Q10
[SURVEY LOGIC – If answering yes to option 3 above (Q9)]
What sources of information did you or your household use when researching dog ownership and/or which breed/crossbreed to buy prior to buying your puppy?
Please select **all options** that apply **[FLIP CHOICES FOR EACH SURVEY TO AVOID BIAS]**
None of these options
The Kennel Club website
An animal charity website, e.g. Dogs Trust, RSPCA, PDSA, etc.
A breed/crossbreed-specific online resource (e.g. website/forum)
Social media sites, e.g. Facebook, Instagram
Book(s)
Dog-specific magazine(s)
My veterinary professional (e.g. veterinary surgeon, veterinary nurse)
Talking to friends or family who own or had owned a dog
Talking to a dog breeder
I can't remember
Other (please specify) **[free text]**

Q11
What characteristics were you looking for in a dog when selecting a particular breed/crossbreed to buy?
Please select **all options** that apply **[FLIP CHOICES FOR EACH SURVEY TO AVOID BIAS]**
I've owned this breed or crossbreed before
I grew up with or had childhood experiences with this breed/crossbreed
Friends or family currently own this breed/crossbreed
Affordable purchase cost of puppies
Affordable cost of upkeep
Appearance/looks
Low grooming needs
Low exercise requirements
Good with children
Good companion
Size suited to my lifestyle
Generally healthy breed/crossbreed
Popularity of the breed/crossbreed
Working ability of the breed/crossbreed
Long life expectancy
Exercise encouragement

Celebrity/Influencer endorsement/ownership
Hypoallergenic
Easy to train
None of these options – I did not have any specific characteristics I was looking for
Other (please tell us here) **[free text]**

Q12
How long after you/your household decided to look for a puppy did you bring your puppy/dog home?
Less than 1 week
Between 1 week-1 month
1 month-6 months
>6 months
I don't remember

Q13
Was the breed or crossbreed of your puppy/dog your first choice?
1. Yes, my/our puppy/dog is the breed/crossbreed that was my/our first choice **[SURVEY LOGIC – skip question 14]**
2. No, I/we could not find a seller that had puppies available at the time for my/our first choice breed/crossbreed
3. No, I/we could not find a breeder I/we felt happy buying a puppy from for my/our first choice breed/crossbreed
4. No, puppies of my/our first choice breed/crossbreed were too expensive
5. No, puppies of my/our first choice breed/crossbreed were too far away
6. Other (please specify) **[free text]**

Q14
[SURVEY LOGIC – If answering anything except for option 1 above (Q13)]
If your puppy/dog is not your first choice of breed or crossbreed, please let us know which breed/crossbreed was your first choice below.
[free text]

Q15
How did you find the breeder of your puppy/dog?
Please select **all options** that apply **[RANDOMISE LIST FOR EACH SURVEY TO AVOID BIAS]**
A general selling website, e.g. FreeAds, Gumtree, Preloved
An animal specific selling website, e.g. Pets4Homes, Champdogs
The Kennel Club website 'Find A Puppy' search
The breeder's website
The breeder's social media account
A social media breed/crossbreed-specific group
Local newspaper advert
Dog specific magazine(s)/newspaper(s)
An advert in a local shop
I already knew the breeder (e.g. colleague, friends, family, repeat purchase)
Recommendation from a friend
Other (please specify) **[free text]**

Q16
What characteristics were you looking for in a breeder?
Please select **all options** that apply **[RANDOMISE LIST FOR EACH SURVEY TO AVOID BIAS]**
Availability of the breed I wanted
Availability of puppies at the time I wanted

Bred the colour of the breed/crossbreed I wanted to purchase
Reasonably priced puppies
Lived within the distance I was willing to travel
Good communication with me
They performed health tests for the breed/crossbreed I wanted
Someone I felt was trustworthy
Someone I felt cared for their dogs
They would allow me to see the puppies' mother
They would allow me to see the puppies' father
They registered their puppies with The Kennel Club
The dogs they bred from had been awarded prizes at dog shows
A member of the Kennel Club Assured Breeder Scheme
Other (please specify) **[free text]**

Q17
Did your breeder question you about your suitability as a dog owner before they agreed to sell you your puppy/dog?
Yes
No
I don't remember

Q18
How much did you pay in total to purchase your puppy/dog?
N.B. Please only include the price of your puppy and not any associated purchases, e.g. food, collar, bowls, etc.
£ (please state a whole number in pounds, do not include the pound sign) **[Number box]**
Prefer not to say
I can't remember

Q19
Where did you/your household go to collect your puppy? [RANDOMISE LIST FOR EACH SURVEY TO AVOID BIAS]

1. The breeder's property – an outdoor kennels, barn or outbuilding
2. The breeder's property – from inside their home
3. The breeder's property – from outside their home, e.g. doorstep, garden
4. A lay-by
5. A car park
6. A service station
7. An airport
8. The breeder delivered my puppy to my property
9. Other (please specify) **[free text]**

Q20
[SURVEY LOGIC – If selecting option 1 or 2 above (Q19)]
What were your/your households first impressions of the environment your puppy was kept in? Please tell us below.
[free text]

Q21
Were you/your household comfortable with the place you received/collected your puppy from?
Yes
No (please explain) **[free text]**

Q22
Did you/your household see your puppy at a date previous to the day you brought them home?
Please select **all options** that apply
Yes – visited the breeder's property in person

Yes – saw my/our puppy on a live video call with their breeder
Yes – saw photos or a pre-recorded video of my/our puppy
No – did not ask to see my/our puppy
No – wanted to see my/our puppy but the breeder refused
No – other (please explain why) **[free text]**

Q23
[SURVEY LOGIC – If yes to option 1 or 2 above (Q22)]
How many times did you/your household see your puppy before you brought them home?
If you did not see them in person or via a live video call, then please enter '0' in that box.
Visits in person **[Number box]**
Live video calls **[Number box]**

Q24
On the day you brought your puppy home, which, if any other dogs did you see your puppy with?
Please select **all options** that apply
Their littermates
Other puppies (unsure if they were littermates)
Their mother
Their father
Another dog(s) they were not related to (e.g. another breed)
I only saw my/our puppy
I don't remember
I'm not sure, I wasn't the person who collected my/our puppy
Other (please specify) **[free text]**

Q25
How old were you told your puppy was when you brought them home?
Under 6 weeks old
7 to 8 weeks old
9 to 10 weeks old
11 to 12 weeks old
13 to 16 weeks old
I'm not sure/can't remember

Q26
Had your breeder provided your puppy with any of the following prior to you taking them home?
Please select **all options** that apply
Worming treatment
Flea treatment
Health check by a vet
Microchip
First vaccinations
Second vaccinations

Q27
Have you been in contact with your breeder since you brought your puppy/dog home?
Yes – I contacted them first
Yes – they contacted me first
No – I tried to contact them but got no response
No – I have not tried to contact them

Q28
Have you registered your puppy/dog with a vet?
1. Yes

2. No
3. No – not yet, but I intend to in the future

Q29
<u>[SURVEY LOGIC – If yes to option 1 above (Q28)]</u>
<u>Have you taken your dog to a vet for any health problems since you brought them home?</u>
No
Yes (please describe the health problem below) **[free text]**

Q30
<u>Has your puppy/dog been vaccinated, or do you plan to in the future?</u>
Yes – just their first vaccinations
Yes – first and second vaccinations
No – not yet, but I plan to in the future
No – not yet, I haven't decided
No – I have chosen not to vaccinate my puppy/dog and don't plan to in the future

Q31
<u>Has your puppy/dog been neutered, or do you plan to have them neutered in the future?</u>
Yes, aged under 6 months
Yes, aged over 6 months
No, but I intend to have them neutered when they are older
No, not yet, I haven't decided
No, but I do not plan to breed from them
No, because I plan to breed from them

Q32
<u>Did you or someone in your household attend any puppy classes with your puppy/dog before they were 16 weeks old?</u>
Yes, in-person puppy classes
Yes, online puppy classes
No, not as yet but I plan to before my puppy is 16 weeks old (if applicable)
No, I wanted to but there weren't any classes running
No, I do not intend to
No, other (please describe here) **[free text]**

Q33
<u>Whilst your puppy was *under 16 weeks* old, did you deliberately leave them alone for any period of time to get them used to being left alone?</u>
Yes
No
No, not as yet but I plan to before my puppy is 16 weeks old (if applicable)
I can't remember

Q34
<u>In hindsight would you change anything about the way in which you acquired your puppy/dog (including your choice of breed and the source you purchased your puppy/dog from)?</u>
<u>Please describe in your own words</u>
[free text]

Q35
<u>Have you considered, or have you needed to, rehome your puppy/dog since you acquired them?</u>
1. I still have my puppy/dog and have not considered rehoming them
2. I still have my puppy/dog, but I have considered, or I am currently considering rehoming them
3. I have rehomed my puppy/dog to another person/family

4. I have given my puppy/dog to a rehoming organisation/charity
5. N/A – My puppy/dog has passed away
6. N/A – My puppy/dog was put to sleep
7. Other (please specify) **[free text]**

Q36
[SURVEY LOGIC – If yes to option 2 or 3 above (Q35)]
If you are comfortable doing so, please describe in your own words why you have rehomed, or thought about rehoming your puppy/dog
[free text]

FILTER:

Q37
1. Yes, I would like to complete more questions, and I bought my puppy home **FROM** 23rd March 2020 onwards **[SURVEY LOGIC – send to Q38 and then to Q66]**
2. Yes, I would like to complete more questions, and I bought my puppy home **BEFORE** 23rd March 2020 **[SURVEY LOGIC – send to Q52 and then to Q70]**
3. No, I do not have time to complete more questions about my puppy's purchase, please take me to the end of the survey (I bought my puppy home **FROM** 23rd March 2020 onwards) **[SURVEY LOGIC – send to Q66]**
4. No, I do not have time to complete more questions about my puppy's purchase, please take me to the end of the survey (I bought my puppy home **BEFORE** 23rd March 2020) **[SURVEY LOGIC – send to Q70]**

2020 EXTENDED QUESTIONS (POST 23/3/2020 OWNERS WITH TIME):

Q38
If you live in a multi-person household, who was the driving force in wanting to acquire a puppy?
Please select **all options** that apply
Myself
Another adult in the household
A child or children in the household
All members of the household were equal in their desire to want a puppy
N/A
Other (please tell us who here) **[free text]**

Q39
Did you feel pressured by your puppy's breeder to commit to buying your puppy?
Yes
No
I'm not sure
I don't remember

Q40
Did you join a waiting list for your puppy?
Yes
No
I don't remember

Q41
After finding your puppy did you put down a deposit to secure him/her?
Yes – before I saw my puppy
Yes – after I saw my puppy
No – I was asked to but refused
No – I was not asked to
I don't remember

Other (please describe here) **[free text]**

Q42

Did your breeder have more than one litter of puppies for sale when you bought your puppy?

Yes – of the same breed/crossbreed

Yes – of more than one breed/crossbreed

No

I'm not sure

I don't remember

Q43

Did your breeder provide you with any of the following items when you collected your puppy?

Column

Yes

No

Not applicable

I'm not sure/can't remember

Row

Puppy's microchip details

Puppy's vaccinations record

Kennel Club change of ownership form

Copy of your puppy's pedigree (family tree)

Food they had been eating at the breeders

Feeding guidance in writing

The Puppy Contract

The Puppy's passport

Q44

Did you ask your breeder to see any information related to health testing of your puppy's parents?

N.B. health tests are not available for all dog breeds

Columns

Yes, and they provided me with it

Yes, but they couldn't provide it

No, I did not ask about this

No, I do not believe there are any tests available for my puppy's breed/crossbreed

Rows

The results of DNA (genetic) tests

The results of veterinary screening tests (e.g. hips, elbows, knees, eyes, respiratory testing)

Q45

Has your puppy's breeder offered you any of the following (at any point in time)?

Please select **all options** that apply

Advice on your puppy's health

Advice on your puppy's training/behaviour

Advice on your puppy's diet

Advice on your puppy's exercise regime

The option to return your puppy to them in the future for any reason

The option to board your puppy with them when on holiday

None of the above

Q46

Soon after you brought your puppy home, did you notice any of the following?

Please select **all options** that apply **[FLIP CHOICES FOR EACH SURVEY TO AVOID BIAS]**

Runny eye(s)

Runny faeces and/or diarrhoea

Being sick (vomiting)

Worms in faeces
Fleas/other parasites visible in fur/on skin
Hair loss
Wounds/sore areas of skin
Frequent itching/licking
Coughing
None of the above
Other (please specify) **[free text]**

Q47
Does your puppy currently have any health issues that you are concerned about?
No
Yes (please specify) **[free text]**
N/A – I no longer have my puppy

Q48
Did your puppy meet any *people from outside your household* between you buying them and reaching 16 weeks of age?
Yes
No
I'm not sure/can't remember
No, not as yet but I plan to before my puppy is 16 weeks old (if applicable)

Q49
Did your puppy meet any *dogs from outside your household* between you buying them and reaching 16 weeks of age?
Yes
No
I'm not sure/can't remember
No, not as yet but I plan to before my puppy is 16 weeks old (if applicable)

Q50
Did your puppy encounter any of the following experiences between you buying them and reaching 16 weeks of age?
Please select **all options** that apply
Column
Yes
No
I'm not sure/can't remember
No, not as yet but I plan to before my dog is 16 weeks old (if applicable)
Row
Walking in a public space (i.e. outside of your home/garden)
Visitors to their home
Fireworks
Thunderstorm
Walking near traffic
Travelling in a car
Dog groomer

Q51
Does your puppy currently show any of the following behaviours that you/your household find problematic?
Please select **all options** that apply **[FLIP CHOICES FOR EACH SURVEY TO AVOID BIAS]**
Pulling on their lead
Jumping up at people
Barking at other dogs
Not coming back when called
Fear of loud sounds (e.g. fireworks, thunderstorms)
Chasing, e.g. cats, wildlife

Clinginess (e.g. following you, sitting close)
Anxiety/fear around other dogs
Anxiety/fear around people in your household (including you)
Anxiety/fear around unfamiliar people
Aggression towards other dogs
Aggression towards people in your household (including you)
Aggression towards unfamiliar people
Guarding of food, toys, or other items
Mouthing
Barking or howling when left alone
Being destructive when left alone
Toileting (weeing or pooing) in the house when left alone
N/A – I no longer have my puppy
None of the above

2019 EXTENDED QUESTIONS (PRE 23/3/2020 OWNERS WITH TIME):

Q52
If you live in a multi-person household, who was the driving force in wanting to acquire a dog?
Please select **all options** that apply
Myself
Another adult in the household
A child or children in the household
All members of the household were equal in their desire to want a dog
N/A
Other (please tell us who here) **[free text]**

Q53
Did you feel pressured by your dog's breeder to commit to buying your dog?
Yes
No
I'm not sure
I don't remember

Q54
Did you join a waiting list for your dog?
Yes
No
I don't remember

Q55
After finding your dog did you put down a deposit to secure him/her?
Yes – before I saw my dog
Yes – after I saw my dog
No – I was asked but refused
No – I was not asked to
I don't remember
Other (please describe here **[free text]**

Q56
Did your breeder have more than one litter of puppies for sale when you bought your dog?
Yes – of the same breed/crossbreed
Yes – of more than one breed/crossbreed
No
I'm not sure
I don't remember

Q57

Did your breeder provide you with any of the following items when you collected your dog?

Column

Yes

No

Not applicable

I'm not sure/can't remember

Row

Dog's microchip details

Dog's vaccinations record

Kennel Club change of ownership form

Copy of your dog's pedigree (family tree)

Food they had been eating at the breeders

Feeding guidance in writing

The Puppy Contract

Dog's passport

Q58

Did you ask your breeder to see any information related to health testing of your dog's parents?

N.B. health tests are not available for all dog breeds

Columns

Yes, and they provided me with it

Yes, but they couldn't provide it

No, I did not ask about this

No, I do not believe there are any tests available for my dog's breed/crossbreed

Rows

The results of DNA (genetic) tests

The results of veterinary screening tests (e.g. hips, elbows, knees, eyes, respiratory testing)

Q59

Has your dog's breeder offered you any of the following (at any point in time)?

Please select **all options** that apply

Advice on your dog's health

Advice on your dog's training/behaviour

Advice on your dog's diet

Advice on your dog's exercise regime

The option to return your dog to them in the future for any reason

The option to board your dog with them when on holiday

None of the above

Q60

Soon after you brought your dog home, did you notice any of the following?

Please select **all options** that apply **[FLIP CHOICES FOR EACH SURVEY TO AVOID BIAS]**

Runny eye(s)

Runny faeces and/or diarrhoea

Being sick (vomiting)

Worms in faeces

Fleas/other parasites visible in fur/on skin

Hair loss

Wounds/sore areas of skin

Frequent itching/licking

Coughing

None of the above

Other (please specify) **[free text]**

Q61
<u>Does your dog currently have any health issues that you are concerned about?</u>
No
Yes (please specify) **[free text]**
N/A – I no longer have my dog

Q62
<u>Did your dog meet any *people from outside your household* between you buying them and reaching 16 weeks of age?</u>
Yes
No
I'm not sure/can't remember

Q63
<u>Did your dog meet any *dogs from outside your household* between you buying them and reaching 16 weeks of age?</u>
Yes
No
I'm not sure/can't remember

Q64
<u>Did your dog encounter any of the following experiences between you buying them and reaching 16 weeks of age?</u>
<u>Please select **all options** that apply</u>
Column
Yes
No
I'm not sure/can't remember
Row
Walking in a public space (i.e. outside of your home/garden)
Visitors to their home
Fireworks
Thunderstorm
Walking near traffic
Travelling in a car
Dog groomer

Q65
<u>Does your dog currently show any of the following behaviours that you/your household find problematic?</u>
<u>Please select **all options** that apply</u> **[FLIP CHOICES FOR EACH SURVEY TO AVOID BIAS]**
Pulling on their lead
Jumping up at people
Barking at other dogs
Not coming back when called
Fear of loud sounds (e.g. fireworks, thunderstorms)
Chasing, e.g. cats, wildlife
Clinginess (e.g. following you, sitting close)
Anxiety/fear around other dogs
Anxiety/fear around people in your household (including you)
Anxiety/fear around unfamiliar people
Aggression towards other dogs
Aggression towards people in your household (including you)
Aggression towards unfamiliar people
Guarding of food, toys, or other items
Mouthing
Barking or howling when left alone
Being destructive when left alone
Toileting (weeing or pooing) in the house when left alone
N/A – I no longer have my puppy

None of the above

2020 COVID SPECIFIC QUESTIONS (POST 23/3/2020 OWNERS WITH AND WITHOUT TIME):

Q66
Had you or someone in your household considered buying a puppy before the COVID-19 pandemic?
Yes
No
I'm not sure

Q67
Do you feel that the COVID-19 pandemic influenced your decision to purchase a puppy?

1. Yes
2. No
3. I'm not sure

Q68
[SURVEY LOGIC – If yes to option 1 above (Q67)]
What were the reasons that the COVID-19 pandemic influenced you/your households' decision to purchase a puppy?
Please select **all options** that apply **[RANDOMISE LIST FOR EACH SURVEY TO AVOID BIAS]**
I/we wanted more company due to being at home more
I/we had more time to care for a dog
I/we wanted more company as family and/or friends were unable to visit me/us
I/we wanted a reason to go outside to exercise more
I/we wanted something happy to focus on
I/we were bored due to the restrictions imposed by lockdown
I/we had extra money to spend that I/we would have usually spent on other things
My child/children were at home and I/we wanted something to keep them busy
Other (please tell us why here) **[free text]**

Q69
When you decided to buy a puppy during the COVID-19 pandemic, did you foresee any additional challenges to owning your puppy in the future (e.g. after restrictions such as lockdown ended)?
Please tell us in your own words below
[free text]

CORE DEMOGRAPHIC QUESTIONS (FOR ALL OWNERS):

Q70
Are you the primary carer for your puppy/dog (i.e. the person in your household that provides your puppy/dog with the majority of care such as feeding and walking)?
Yes
No
I share the role of primary carer for my puppy/dog with someone else in the household
I share the role of primary carer for my puppy/dog with someone else in a different household
N/A – I no longer have my puppy/dog

Q71
How old are you?
18 to 24 years old
25 to 34 years old
35 to 44 years old
45 to 54 years old

55 to 64 years old
65 to 74 years old
75 years old or older

Q72
<u>What is your gender?</u>
Female
Male
Other
Prefer not to say

Q73
<u>If you are happy to, please provide the first three/four digits of your postcode (e.g. AL9)</u>
[free text]

Q74
<u>Were you/your household affected by the COVID-19 pandemic in any of the following ways?</u>
<u>Please select **all options** that apply</u>
One or more members of my household/I was furloughed
One or more members of my household/I started working from home
One or more members of my household/I became unemployed
Child(ren) had to be homeschooled or cared for from home
None of the above
Prefer not to say

Q75
<u>Were you or any member of your household classed as a key worker during the COVID-19 lockdown?</u>
<u>Please select **all options** that apply</u>
Yes, I am a key worker
Yes, another member of my household is a key worker
No
I prefer not to say
I’m not sure

Q76
<u>Are you or any member of your household employed in the canine and/or animal care sector (e.g. veterinary nurse, dog groomer, dog trainer, etc.)?</u>

1. Yes
2. No
3. I’m not sure

Q77
<u>**[SURVEY LOGIC – If yes to option 1 above (Q76)]**</u>
<u>Which canine and/or animal care sector are you or a member of your household employed in?</u>
<u>Please select **all options** that apply</u> **[RANDOMISE LIST FOR EACH SURVEY TO AVOID BIAS]**
Veterinary surgeon
Veterinary nurse
Animal care assistant
Veterinary scientist
Dog behaviourist
Dog trainer
Dog daycare/boarding kennels
Dog walker
Dog groomer
Rehoming center staff

Other (please specify) **[free text]**

Q78
Did you grow up with a dog in your childhood home?
Yes
No

Q79
As an adult, have you ever owned or co-owned a dog before you purchased your puppy/dog?
Yes
No – but someone else in my household has
No – I am/everyone in my household is a first-time dog owner(s)

Q80
What best describes your current living situation?

1. Live alone
2. Live in an adult only home (over 18 years old)
3. Live in a home with adults and children
4. Live in a home with children where I am the only adult
5. Other (please specify) **[free text]**

Q81
[SURVEY LOGIC – If yes to option 3 or 4 above (Q80)]
How old are the children that your dog/puppy currently shares a home with?
Please select **all options** that apply
Under 5 years old
5-10 years old
11-15 years old
16-18 years old

Q82
How many (if any) other dogs currently live in the same home as your puppy/dog?
Please enter a whole number
N.B. If they are the only dog in your household, please type '0'
[Number box]

Q83
Does your puppy/dog currently have access to outdoor space?
Yes – a garden or yard (private)
Yes – a garden or yard (shared)
No
N/A – I no longer have my puppy/dog

Q84
Is your puppy/dog *currently* left at home alone for more than four hours without being taken out for exercise or having someone come in to check on them?
Yes
No
I'm not sure
N/A – I no longer have my puppy/dog

Q85
In *the future*, is your puppy/dog likely to be left at home alone for more than four hours without being taken out for exercise or having someone come in to check on them?
Yes
No
I'm not sure
N/A – I no longer have my puppy/dog

Q86
How much do you expect your puppy/dog to cost per year? Please consider all ongoing costs, for example food, equipment and veterinary bills for preventative healthcare such as worming (but NOT illness/injury).
£ (please state as a single number, not a range) **[Number box]**
I'm not sure
N/A – I no longer have my puppy/dog

Q87
We may wish to get in contact with you in the future for limited reasons outlined below.
Please let us know which (if any) you are happy to be contacted about, and provide us with your preferred email address:
To be asked to clarify my responses to this study
To be sent the results of this study
To be invited to take part in further research about my puppy/dog
None of the above
Comment box: **[email address]**

DEBRIEF QUESTIONS (ALL OWNERS):

Q88
Had you heard of the Petfished Campaign before purchasing your puppy?
Yes
No

Q89
Had you heard of The Puppy Contract before purchasing your puppy?
1. Yes
2. No

Q90
[SURVEY LOGIC – If yes to option 1 above (Q89)]
Did you use The Puppy Contract when purchasing your puppy?
If you did not on this occasion, please explain in your own words why you chose not to or were unable to
Yes
No (please describe why) **[free text]**

Qualitative Coding Framework for Content Analysis

Generic coding rules

Back Allocating to Existing Deductive Category:
Free-text responses were allocated to the existing deductive categories (MCQ choices) if not selected by the respondent.

Miscellaneous free-text response:
Free-text responses which did not explicitly answer the question were classified as 'Miscellaneous' and were not included in the analysis.

Uninterpretable:
Free-text responses consisting of meaningless digits or punctuation were classified as 'uninterpretable' and not included in the analysis.

Creating New Inductive Categories:
Where comments did not correspond to the existing deductive categories (MCQ choices), new categories were created as explained in the accompanying paper (section 2.5. Qualitative Content Analysis of Free-Text Options).

In the case of questions where only one response was requested, the first comment in the free-text was either assigned to a new category or back allocated to an existing category. For questions where multiple responses were allowed, all relevant comments were assigned to existing category(s) and/or assigned to new category(s).

The following tables give details of both the existing and new categories per question analysed for this publication, along with examples of comments for each.

Q4: Is your puppy/dog registered with The Kennel Club?

- No – registered with another canine registration body, e.g. a working dog registry (please specify) (n=68)

Back Allocating to Existing Deductive Category	Example(s) Back Allocated
Yes	*"Duel reg international sheep dog society and KC"*
Not applicable – crossbreed	NONE
Not applicable – breed not registered with The Kennel Club	*"Working Kelpie Council of Australia", "Tamaskan dog register", "Koolie Registry"*
I'm not sure	*"Can't remember"*
No	*"No, British bulldog association"*

New Category	Example(s)
KC Activity Register	*"Kc activity registered", "K C working register for competitive obedience"*
International Kennel Club	*"FCI", "Canadian Kennel Club", "ASCA - Australian Shepherd Club of America"*
Working Dog Registry	*"ISDS", "Working Kelpie Council of Australia", "Duel reg international sheep dog society and KC"*
Non-KC UK Registration Body	*"NEBBR", "Dog world kennel club"*
Miscellaneous	*"Pup imported, registered in country of origin", "Local council"*

Q7: Is your puppy/dog insured?

- No – other (n=31)

Back Allocating to Existing Deductive Category	Example(s) Back Allocated
Yes	NONE
No – and I do not plan to insure them	*"4 weeks insurance, will not renew"*
No – but I plan to insure them in the future	NONE
No – they were insured but I have since cancelled or did not renew their policy	NONE
No – I have never heard of pet insurance	NONE
Yes	*"Came with 5 weeks insurance and will continue with our own insurance when it runs out."*

New Category	Example(s)
Yes, under free insurance from the breeder and undecided whether to continue	*"She came with 4 weeks free insurance. I am currently looking into insurance to see if it will be worthwhile."*
N/A, we no longer have our puppy	*"Puppy was fully insured but died.", "Puppy was insured but died five days after I bought him so insurance was cancelled"*
Miscellaneous	NONE

Q8: What were the main reasons your household wanted to acquire a dog?

- Other reason not listed above (n=979)

Back Allocating to Existing Deductive Category	Example(s) Back Allocated
Companionship for myself	*"Companionship, though I am not sure whether this is the right description for wanting a dog…", "…I could tick several of the boxes regarding companionship, working dog, mental health etc."*
Companionship for my children	*"As a benefit for the whole family, especially child going through pandemic exams", "For my children's enjoyment", "For children to experience having a family pet…", "The children wanted him"*
Companionship for other adult(s) in my household	*"All 4 adults in our home just love having a dog around the house. Our puppy gives us companionship and love."*
Companionship for my other dog(s)	*"As a companion for my other Italian Greyhound."*
To keep me/my family busy	*"Family pet to share responsibilities with young teenagers, support communication and thinking if others ie the dog"*
To encourage myself/my family to walk and exercise	*"A dog that could walk in the mountains with us.", "Greater enjoyment of walks…"*
To improve my/my family's mental health	*"…the joy a dog can bring to family life", "Joy. Love."*
Due to the loss of a previous dog in my household	*"I've always had 2 dogs list one of them so got my old dog a playmate", "We lost our dog 1.5 years ago and it was time to get another"*
As a working dog for a specific role (e.g. gundog, security, sniffer/tracking, herding, medical detection, assistance/therapy dog)	*"Assistance dog for Autistic child", "Emotional support dog for child", "Hopefully later as an epilepsy sensitive dog.", "I want to get into search and rescue voluntary work with my dog", "Security"*

New Category	Example(s)
For a specific non-working role (e.g. dog sports, showing, etc.)	*"A second agility dog", "As a show dog", "Sport dog - canicross and mantrailing"*
For breeding (including stud dogs)	*"Breeding, already have 2 dogs and what to breed one of them.", "Breeding", "Potential stud"*
Due to the ageing of another dog/other pet in my household	*"Older dog diagnosed with cancer", "My old dog is now 14yo and will likely pass away within the next year, so I was looking to get a replacement...", "Old dog is getting too old for the long walks that we do as a family"*
Companionship for my other (non-dog) pets	*"Company for my cat who has always lived with a dog…", "Friend for my cat"*
Due to the death of another (non-dog) pet in my household	*"Cat died", "After the loss of our cat we missed having an animal in our house", "Always wanted a dog but didn't as we had a rabbit, when the rabbit died we decided to get a dog", "Have intended to get a dog for a long time. Losing our chickens to a fox meant we were now able to do this."*
Miscellaneous	*"Always wanted a dog however due to work was not able to give it attention a puppy would require. Lockdown gave us the time we needed"*

Q10: What sources of information did you or your household use when researching dog ownership and/or which breed/crossbreed to buy prior to buying your puppy?

- Other, please specify (n=497)

Back Allocating to Existing Deductive Category	Example(s) Back Allocated
The Kennel Club website	NONE
An animal charity website, e.g. Dogs Trust, RSPCA, PDSA, etc.	NONE
A breed/crossbreed-specific online resource (e.g. website/forum)	*"Breed club websites", "Breed specific internet sites", "Dachshund breed council website", "Websites dedicated to the breed"*
Social media sites, e.g. Facebook, Instagram	*"Mumsnet "The Doghouse" forum", "…YouTube for training videos…", "YouTube…"*
Book(s)	NONE
Dog-specific magazine(s)	NONE
My veterinary professional (e.g. veterinary surgeon, veterinary nurse)	*"I am an RVN so spoke to friends who are RVN's & vets", "…a friend's dad who is a vet"*
Talking to friends or family who own or had owned a dog	*"I contacted friends and peers within the breed", "My dad", "My family always had dogs when I was growing up"*
Talking to a dog breeder	*"Also spoke to breeder direct..."*
None of these options	NONE
I can't remember	NONE

New Category	Example(s)
I am already an experienced dog owner but purchased a new breed	*"Drew on own lifelong experience then lots of online owners clubs", "I have all ways had dogs, it was only the breed I needed information on."*
Seeking practical experience of caring for dogs (e.g. Borrow My Doggy/dog sitting, fostering, etc.)	*"…We have been dogsitting for friends on holiday etc in years previous to purchase", "Borrow my Doggy", "Dog sitting for friends to try out experience", "Experience as a volunteer for Hearing Dogs for Deaf People"*
Talking to a non-veterinary animal professional (e.g. trainer, behaviourist)	*"…Gundog Trainer", "Dog behaviourist / trainer", "Dog groomer", "Local dog trainer", "Pet shops…", "Talking to dog behaviourists"*
I have professional experience with dogs (veterinary and non-veterinary)	*"Am a vet", "As a professional dog trainer I knew what I wanted.", "Having various breeds to stay whilst running a home from home dog boarding business", "I am a veterinary behaviourist", "Myself (I am a vet)"*
Talking to current dog owners that I met (not friends or family)	*"Visited the breed section at crufts to see examples of the breed and speak to owners", "Talking to owners of dogs I liked at competitions…", "Talking to other dog owners we didn't know but met when out."*
Other digital media sources (internet searches/sites, TV, DVD, etc.)	*"Articles on the internet", "General internet searches", "Google", "Pets4Homes breed specific information.", "TV shows/documentaries", …Dog training programmes such as Dogs Behaving Badly."*
Miscellaneous free-text response	*"Lots of people telling you what not to do and what to avoid very little positive help ended up using my own instincts", "We were limited to availability because of Covid and extortionate prices"*

Q11: What characteristics were you looking for in a dog when selecting a particular breed/crossbreed to buy?

- Other, please tell us here (n=558)

**Back Allocating to Existing Deductive Category**	**Example(s) Back Allocated**
I've owned this breed or crossbreed before	*"Already own a bulldog", "His predecessor was also a whippet", "I already own a 9 year old Jack Russell"*
I grew up with or had childhood experiences with this breed/crossbreed	*"A relative had 2 when I was a child.", "Grew up with springers…"*
Friends or family currently own this breed/crossbreed	*"Friends have this breed and I grew very fond of their dog,"*
Affordable purchase cost of puppies	*"…they seemed to be lower priced than they had been before"*
Affordable cost of upkeep	NONE
Appearance/looks	*"She is a blue girl, like her great great grandmother"*
Low grooming needs	*"…short haired…"*
Low exercise requirements	*"Reasonable exercise requirements...",*
Good with children	NONE
Good companion	*"…family companionship"*
Size suited to my lifestyle	*"Size", "I just wanted a nice dog of a reasonable size", "Suitable breed size with an elderly parent round…"*
Generally healthy breed/crossbreed	*"Wanted a Labrador but have an older Labrador with health concerns, was hoping a cross breed may be healthier."*
Popularity of the breed/crossbreed	NONE
Working ability of the breed/crossbreed	*"Working ability", "specifically for picking up on small shoots (but a pet first)"*
Long life expectancy	NONE
Exercise encouragement	*"Exercise wanted a dog that was able to walk/ run long distances", "Active dog, could join family on long walks"*
Celebrity/Influencer endorsement/ownership	NONE
Hypoallergenic	*"Hypoallergenic as my husband has asthma and is allergic to cats", "…unlikely to cause allergies…"*
Easy to train	*"…trainable…", "Trainability", "…easy to train."*
None of these options – I did not have any specific characteristics I was looking for	*"None of these, the puppy became available through a friend's dog becoming unexpectedly pregnant", "…we weren't looking specifically but it came up in conversation that she was available…"*

**New Category**	**Example(s)**
None of these options – someone else in the household selected the breed/crossbreed of our puppy	*"Daughter wanted this breed and I wanted her to be involved", "Husbands preference…", "My son has always wanted this breed.", "Was a breed my partner had always loved since a child"*
Other perceived temperament/personality traits of the breed/crossbreed	*"A dog with personality. Perhaps a challenge.", "A good personality", "…Good natured with dogs and humans", "…Wanted a dog with a low chance of being aggressive towards other dogs if trained correctly…"*
Low or non-shedding breed/crossbreed	*"Did not moult", "Doesn't malt", "…one with less shedding", "Low shedding", "Non-shedding"*
Specific genetic characteristics of the breed/crossbreed	*"To maintain my breeding lines", Specific breeds and lines for what we want", "Working ability of these lines…"*
I've always wanted to own this breed/crossbreed	*"…always loved this breed. I've wanted to own one since childhood.", "Wanted one since I was a kid"*
Miscellaneous free-text response	*"Came from a home we knew well", "Grew up in a family that always had pets including dogs"*

Q13: Was the breed of your puppy/dog your first choice?

- Other (please specify) (n=376)

NOTE: Only one response was requested for this question and as such just the first comment in the free-text was assigned to a single new category or back allocated to a single existing category.

**Back Allocating to Existing Deductive Category**	**Example(s) Back Allocated**
Yes, my/our puppy/dog is the breed/crossbreed that was my/our first choice	*"Yes because we knew someone who already owned a related dog to the puppy that we bought", "Yes but we couldn't afford a KC registered puppy, so had to choose a non-KC registered one…", "Breed of choice but researched many breeders and followed their dogs for over 18months before deciding on a specific breeder and litter", "First choice breed and parents…"*
No, I/we could not find a seller that had puppies available at the time for my/our first choice breed/crossbreed	*"Due to get another breed in May but took the opportunity to get a puppy sooner having waited almost 2 years and our wedding not going ahead this year", "no - breeder had sold the breed we enquired but had shihpoo's available"*
No, I/we could not find a breeder I/we felt happy buying a puppy from for my/our first choice breed/crossbreed	*"Could not find a breeder I was happy with AND the price went up exponentially", "Originally wanted a Labrador but could not find a breeder I trusted completely…", "We looked into several puppies but something just didn't feel right as in they seemed to be too much of a frequent breeder…"*
No, puppies of my/our first choice breed/crossbreed were too expensive	*"No - we placed a deposit for a cavapoo before the pandemic but once we moved into lockdown our dog doubled in price with explanation from the breeder.", "Initially we were looking at a Jack Russell puppy however the breeder put their prices up by £500 due to the pandemic and changed the agreed price so I started looking at other breeds."*
No, puppies of my/our first choice breed/crossbreed were too far away	NONE

**New Category**	**Example(s)**
No, I/we wanted a rescue dog but were unable to source one	*"Couldn't find a suitable rescue pup for our lifestyle", "I had looked at adopting rescue dog but could not find one and then friend encouraged me to look at puppies", "I tried to rescue a dog for many months prior to buying"*
No, I/we didn't have a specific choice in mind	*"Breeder was known to a friend, and I was not set on a particular breed.", "Didn't have a preference"*
No, I/we changed our mind	*"No. Initially I was looking at a malshi but after more research realised a flat was not ideal", "Changed our minds", "First choice was a golden retriever but son was allergic so we met with the breeder of the Goldendoodle"*
No, our planned purchase of our first choice fell through	*"Had arranged with a breeder to have a dog but sadly all the pups died so we had to go elsewhere", "No, I was going to get a Border Terrier puppy and paid a deposit. Sadly I was the victim of a scam and the breeder took my money and then ceased all communication. I therefore did not get the puppy I initially chose. Border Terriers were then too expensive so I researched other breeds and found my Shih Tzu"*
No, but I had up to three breeds I was interested in and got one of those	*"Equally wanted either working bearded collie or bearded collie cross border collie.", "We had a shortlist of 3 breeds we preferred, we couldn't find any puppies of the other breeds at the time we were ready to buy"*
No, but I had more than three breeds I was interested in and got one of those	*"I considered a beagle, a beaglier, King Charles or a puggle...I didn't mind which", "I wanted a small poodle mix, I was open to several crossbreeds"*
Miscellaneous free-text response	*"We would have liked a different colour but couldn't get one locally", "I wasn't sure I wanted the commitment of a puppy after losing our dog 4 months previous"*

Q15: How did you find the breeder of your puppy/dog?

- Other (please specify) (n=504)

Back Allocating to Existing Deductive Category	Example(s) Back Allocated
A general selling website, e.g. FreeAds, Gumtree, Preloved	*"Free ads", "Gumtree"*
An animal specific selling website, e.g. Pets4Homes, Champdogs	*"Also looked at champ dogs…", "Contacting the breeder via details saved on the ChampDogs website…", "Pets4Home", "Pets4Homes website"*
The Kennel Club website 'Find A Puppy' search	*"Kennel Club website to find the breeder then called the breeder to find out more"*
The breeder's website	NONE
The breeder's social media account	*"Instagram", "Followed the breeder via their Facebook page for over 1 year…"*
A social media breed/crossbreed-specific group	*"…following fb pages etc"*
Local newspaper advert	NONE
Dog specific magazine(s)/newspaper(s)	NONE
An advert in a local shop	NONE
I already knew the breeder (e.g. colleague, friends, family, repeat purchase)	*"A friend bred her.", "A friend dogs just breed one day", "Already knew the breeder", "Client at work", "Family friend", "Knew the breeder"*
Recommendation from a friend	*"Found through agility contacts, friend of a friend"*

New Category	Example(s)
Recommendation from another breeder/stud dog owner	*"A breeder recommended their friend who was hoping to breed her dog."*
Recommendation from someone who is not a colleague, friend, family or animal professional	*"recommendation from breed club secretary", "…via the local council approved breeder list…", "Breed Club", "Crufts Discover Dog's", "Crufts"*
Recommendation from someone who is an animal professional (e.g. veterinary surgeon, veterinary nurse, dog trainer, etc.)	*"A friend who is also a vet referral physio", "Recommendation from my vet", "Referred by a trainer", "Vet recommended"*
Recommendation from a stranger after a chance encounter	*"We had a chance encounter with a couple in the Lake District who had a lovely cockapoo, when we started talking to them, we found that the breeder of their dog was expecting another litter. We took the breeder's contact details and called them to enquire."*
An advert seen in another online location (including general internet searches and social media)	*"Google search", "Googled puppies for sale within a set radius from our home", "Local town social media account.", "Online research", "On our village Facebook Page, it was fate!", "Vlog on YouTube"*
A physical advert in another location (e.g. vets noticeboard)	*"Sign outside the farmers driveway.", "notice on the vets' notice board seemed a good place to start", "Advertised in the vets"*
The breeder contacted me directly following expression of interest for a puppy	*"Breeder sought me out after she saw me enquiring on Facebook", "I put an advert that we were looking on pets4homes and the breeder contacted me…"*
Miscellaneous free-text response	*"Also saw the breed in 101 dalmations"*

Q16: What characteristics were you looking for in a breeder?

- Other (please specify) (n=553)

**Back Allocating to Existing Deductive Category**	**Example(s) Back Allocated**
Availability of the breed I wanted	*"Bred the type of Labrador I liked.", "The breeding lines that I wanted a pup from."*
Availability of puppies at the time I wanted	NONE
Bred the colour of the breed/crossbreed I wanted to purchase	NONE
Reasonably priced puppies	*"…They priced their pups at normal prices pre pandemic and not the current extortionate amounts"*
Lived within the distance I was willing to travel	*"…and lived near me"*
Good communication with me	NONE
They performed health tests for the breed/crossbreed I wanted	NONE
Someone I felt was trustworthy	*"Honesty, openness, willing to answer all questions,"*
Someone I felt cared for their dogs	*"Felt like they interviewed me which showed they cared about the puppies welfare"*
They would allow me to see the puppies' mother	NONE
They would allow me to see the puppies' father	NONE
They registered their puppies with The Kennel Club	NONE
The dogs they bred from had been awarded prizes at dog shows	NONE
A member of the Kennel Club Assured Breeders Scheme	NONE

**New Category**	**Example(s)**
A council registered breeder	*"5 star rated licenced breeder", "Breeder registered with Council", "Registered as a breeder with Local Authority"*
Someone I already knew	*"We had bought from them before..", "Same parents as existing dog", "Knew the breeders"*
They bred dogs with specific working and/or sporting characteristics	*"A line of springer that did not have rytex in it as i wanted a old fashion springer for work", "Bred the puppies from a good working line and the parents were proven working dogs", "…breeder well known in sheepdog trialling for producing good working dogs", "Someone who raced their whippets and had proven success"*
They were a member of a specific breed/crossbreed club/association	*"…Puppy was ISDS registered…", "Member of breed club", "founder member of the labradoodle club.", "Dog was registered with NEBBR…", "Active member of Bernese Mountain Dog Association"*
They registered their puppies an international canine registration body (e.g. FCI)	*"Fci breed club member. Fci registered puppies…", "Registered with Italian Kennel Club (is an Italian)", "…FCI registered breeder…"*
Miscellaneous free-text response	*"I'm a teacher so we wanted to bring a puppy home when I would be there to devote time to initial settling in."*

Q19: Where did you/your household go to collect your puppy?

- Other (please specify) (n=441)

NOTE: Only one response was requested for this question and as such just the first comment in the free-text was assigned to a single new category or back allocated to a single existing category. The key consideration with this question was to consider the final destination where the owner took possession of their puppy, not where it came from.

Back Allocating to Existing Deductive Category	Example(s) Back Allocated
The breeder's property – an outdoor kennels, barn or outbuilding	*"…The pups were in a kennel outside in the garden…", "The breeder had set up a special shed at the front of her property due to covid restrictions…"*
The breeder's property – from inside their home	*"Inside their home, we visited several times"*
The breeder's property – from outside their home, e.g. doorstep, garden	*"At the breeders house, due to lockdown exchange was outside…", "Breeders property in their field", "Chose our puppy in breeders garden, because of covid 19.", "…we 'exchanged' on their doorstep…"*
A lay-by	*"…I collected from a road as a long drive away, and wanted to get pup home asap"*
A car park	*"Due to travel restrictions on the ferry to Arran we had to meet in a car park.", "…Due to the distance (they were over 500 miles away) we picked up the puppy from the car park at a show that was midway between us"*
A service station	*"Met my breeder halfway at a service station", "…We picked her up from a services as agreed with our breeders…"*
An airport	NONE
The breeder delivered my puppy to my property	*"…they dropped her off as they were driving within 5 miles of the house on their way to a family holiday"*

New Category	Example(s)
The breeder's non-residential property (e.g. holiday home) or workplace	*"A stable yard owned by the breeder", "At her place of work", "Breaders grooming shop", "Breeder's holiday home as she said the home had an outdoor space at the front where we could safely see the puppies instead of her home", "On investigation by the SSPCA, the address given as the breeders turned out to be an AirBNB which they had rented for the purpose of selling farmed puppies"*
A veterinary practice	*"We met her at the centre where the litter were being tested by a vet for eye problems", "…We took our puppy at a health check appointment at the vet as the breeder wanted us to be part of that visit.", "Veterinary surgery"*
Another transport station/port (e.g. train station, ferry port)	*"Dog was transported from Hungary and I met the transport at the Channel tunnel", "Ferry Port - the breeder drove with several puppies from NI and new owners met them at the port."*
Another public location (e.g. a park, hotel, a shop)	*"…They traveled from Belgium with their dogs for Crufts and my puppy, met them at their hotel…", "A park"*
Breeder/courier met us halfway (location not specified)	*"As it was lockdown they met us half way as they were dropping another puppy at their new home", "As We went into lockdown when ready to come home we met halfway"*
Courier/pet transporter to my property	*"…breeder organised for puppy to be delivered to us by pet courier which we paid for on top of purchase price.", "We used a puppy transporter as it was lockdown.", "Pet transporter imported the puppy at 15 weeks old", "Puppy traveled from the breeders house in Russia by a mutually agreed courier directly to my house", "Due to lockdown, the breeder arranged professional transport for the puppy"*
My friend collected and delivered my puppy for me	*"Friend collected them on way back from work…", "My friend collected hers and my puppy as they wasn't local"*
Miscellaneous free-text response	*"We wore masks & socially distanced when we visited & when we collected our puppy"*

Q22: Did you/your household see your puppy at a date previous to the day you brought them home?

- Other (please explain why) (n=681)

**Back Allocating to Existing Deductive Category**	**Example(s) Back Allocated**
Yes – visited the breeder's property in person	*"I had called to do a first visit. I was satisfied it was not a puppy farm and the dog appeared healthy and energetic and mum looked healthy. The farmer showed off some of her obedience training."*
Yes – saw my/our puppy on a live video call with their breeder	*"Because of lockdown we could not view the puppy, but we did have regular video calls with the breeder", "Due to COVID restrictions we could not meet him beforehand so had FaceTime calls with the breeder"*
Yes – saw photos or a pre-recorded video of my/our puppy	*"Only in pictures", "…We asked for weekly updates & photo's, we only received 4 before getting him.", "Saw photos. Breeder lived 6h drive away so prior visit not possible"*
No – did not ask to see my/our puppy	*"She had only advertised them the week before we went to visit following weekend, a few bitches had been reserved but we wanted a dog. He was already 8 weeks old so we came away with him on the grounds of a health check being ok with our own vets if not he would be returned", "We did not see this puppy before purchase as it was a bit of an impulse decision…"*
No – wanted to see my/our puppy but the breeder refused	*"We lived far away and had asked multiple times for videos/pictures and the breeder kept saying they'd get round to it but never did.", "Breeder continually said we couldn't visit puppy as he was shielding a vulnerable partner…", "Breeder initially wouldn't allow it because of lockdown but then as restrictions started to ease would not respond to any messages where we asked to come and see the puppy…"*

**New Category**	**Example(s)**
No – but a friend/relative visited the breeder's property on my behalf	*"Because of the distance a friend who lived closer called for me to view home as much as pups", "…my friend (who lived locally to the breeder) visited & also sent me pictures"*
No – purchasing my puppy was a rapid decision and I brought my puppy home on the same day as viewing	*"It was 150 mile drive and had been trying to find a puppy for some time, so felt under pressure to find and buy one ASAP.", "I found the puppies at 7.5 weeks old, we were on our way to visit and the breeder called during our journey to say we could take her on the day as we were travelling 4 hours and she's weaned and very close to 8 weeks."*
No – I asked to visit but personal circumstances meant I was unable to visit myself or get someone else to visit on my behalf	*"Arranged to visit but couldn't get the time off work", "I was ill when we were meant to visit", "it wasn't possible as we were on holiday"*
No – I was unable to visit due to the breeder being too far away to travel to	*"Breeder lived 550 miles away!...", "…Was invited to see pup before purchase but we lived too far away.", "Distance meant I couldn't.", "It was 300 miles away"*
Yes – I saw my puppy in person but not at the breeder's property	*"I am a veterinary nurse and the breeder was a client of ours she has bought int he lotter for first vaccinations…", "At her work place"*
No – I only spoke to the breeder by telephone	*"Our breeder is not tech savvy, so wasn't able to video call and only spoke on phone…", "…We spoke on the phone and i very strongly felt that she was a lovely kind person."*
Miscellaneous free-text response	*"We collected the puppy the day before lockdown.", "They did seem to care greatly for the pups"*

Q24: On the day you brought your puppy home, which, if any other dogs did you see your puppy with?

- Other (please specify) (n=742)

**Back Allocating to Existing Deductive Category**	**Example(s) Back Allocated**
Their littermates	NONE
Other puppies (unsure if they were littermates)	NONE
Their mother	NONE
Their father	NONE
Another dog(s) they were not related to (e.g. another breed)	NONE
I only saw my/our puppy	NONE
I don’t remember	NONE
I’m not sure, I wasn't the person who collected my/our puppy	NONE

**New Category**	**Example(s)**
Another adult dog(s) they were related to (e.g. aunts, grandparents, older siblings)	*“Other related dogs - grandmother and aunt of the puppies.”*, *“Plus the mother's puppy from an earlier litter”*, *“Puppy’s grandmother and uncle”*
Another adult dog(s) the breeder claimed were my puppy’s parent(s), but I’m not sure	*“Breeder was dodgy so unsure whether we saw the mum…”*, *“In not convinced they were his actual parents”*, *“Now not convinced it was the mother”*
Other puppies they were related to (but not littermates)	*“Separate related litter”*
Other puppies they were not related to (e.g. another breed)	*“…another litter that was not related”*
Miscellaneous free-text response	*“Another person was collecting just as we arrived but they had staggered times”*, *“Because of lock down we could only collect puppy from owners garden.”*, *“Cats”*, *“Chickens and Cockerel”*

Q38/Q52: If you live in a multi-person household, who was the driving force in wanting to acquire a dog/puppy?

- Other (please tell us who here) (n=69)

Back Allocating to Existing Deductive Category	Example(s) Back Allocated
Myself	*"Me."*
Another adult in the household	*"I had cats growing up and would have happily had a pet cat, but my partner has always wanted a dog. He persuaded me that we should get a dog…"*
A child or children in the household	*"Younger son"*
All members of the household were equal in their desire to want a dog	NONE
N/A	*"I live alone", "I live in single person household so just me"*

New Category	Example(s)
The majority of members in the household	*"4 members were for and 1 against", "All except my husband!", "Husband and I and 2 of our 3 children wanted a dog!"*
Another family member who lives outside the household	*"Visiting toddler and small grandchildren", "My parents (who live elsewhere) also wanted to help with a pup after losing their dog", "My elder daughter who does not live with us shares the expense and walks her at weekends"*
Miscellaneous free-text response	*"Would of prefered to take on a rescue dog of this breed, but have been on a waiting list for 4 years. No problem with us, have had centre ask us in past to take on dogs. With covid I can't see it getting any better, so we decided on a puppy."*

Q41/Q55: After finding your dog/puppy did you put down a deposit to secure him/her?

- Other (please describe here) (n=255)

**Back Allocating to Existing Deductive Category**	**Example(s) Back Allocated**
Yes – before I saw my puppy	*"£50 before puppy was conceived.", "100 refundable deposit before seeing him…", "Had to give a deposit before the puppy was born"*
Yes – after I saw my puppy	*"Yes after I saw my puppy via video call and saw the mother on video call from the breeders family home", "After a zoom call"*
No – I was asked to but refused	NONE
No – I was not asked to	*"Actually I offered but was told not required", "As I saw the puppy only a week before I brought it home, I was not asked to.", "I knew the breeder so was not required to like the other people purchasing puppies from the same litter"*
I don't remember	NONE

**New Category**	**Example(s)**
No – there was no time as I visited, paid for and took my dog home on the same day	*"No, pup was available to collect when first saw advertisement. Pup collected same day"*
Miscellaneous free-text response	NONE

Q68: What were the reasons that the COVID-19 pandemic influenced you/your household's decision to purchase a puppy?

- Other (please tell us why here) (n=579)

**Back Allocating to Existing Deductive Category**	**Example(s) Back Allocated**
I/we wanted more company due to being at home more	*"…I wanted to have a dog for company as I live alone…"*
I/we had more time to care for a dog	*"Working from home so had the time", "Working from home meant we could care for a puppy", "…as my husband was furloughed we decided to get the puppy sooner as we would have more time together to get the puppy settled."*
I/we wanted more company as family and/or friends were unable to visit me/us	NONE
I/we wanted a reason to go outside to exercise more	*"… getting family outdoors…", "…give us more reason to go out for walks as a family…"*
I/we wanted something happy to focus on	*"…It would also be something positive to focus on…", "…We got our pup to help with our grieving and give us some joy in this sad time"*
I/we were bored due to the restrictions imposed by lockdown	NONE
I/we had extra money to spend that I/we would have usually spent on other things	*"Our cruise got cancelled so we used some of the money"*
My child/children were at home and I/we wanted something to keep them busy	NONE

**New Category**	**Example(s)**
My/our ability to acquire a dog/puppy from another source was affected by the COVID-19 pandemic so we bought a puppy instead	*"We were looking at older rescues but none were available…", "We wanted to adopt a rescue, but they were inundated with applications and we didn't even get to the homecheck stage.", "Restrictions on rescue charities as well as increased demand made this route to dog ownership difficult."*
I/we experienced mental health challenges due to the COVID-19 pandemic that I/we wanted a puppy to help us with	*"I was in the house every day by myself and it had a bad effect on my mental health…", "…I was unhappy and very depressed and had to live in total isolation because of COVID. I was worried about my emotional state and felt having a dog depend on me would be beneficial"*
My/our existing dog/pet experienced mental health challenges as a result to the COVID-19 pandemic that I/we wanted a puppy to help them with	*"My other dog started to suffer anxiety, when I left the house, as I was at home so much. I had planned on getting a second dog next year but brought it forward because of this."*
Miscellaneous free-text response	*"We lost our chickens to a fox and therefore could have a puppy (after discussing it for several years)"*

Q77: Which canine and/or animal care sector are you or a member of your household employed in?

- Other (please specify) (n=205)

Back Allocating to Existing Deductive Category	Example(s) Back Allocated
Veterinary surgeon	NONE
Veterinary nurse	NONE
Animal care assistant	NONE
Veterinary scientist	NONE
Dog behaviourist	NONE
Dog trainer	NONE
Dog daycare/boarding kennels	*"Home boarder and daycare"*
Dog walker	NONE
Dog groomer	NONE
Rehoming centre staff	NONE

New Category	Example(s)
Academic sector (e.g. university staff and students)	*"…university student studying Animal Behaviour", "Animal care lecturer", "Current vet student", "Lecturer in clinical animal behaviour…"*
Allied veterinary professionals (e.g. physiotherapist, hydrotherapist)	*"…Canine Nutritionist for a dog food company", "Radiographer at a referral hospital", "Receptionist at vet hospital", "Veterinary physiotherapist", "Canine Hydrotherapist"*
Animal care sector (e.g. pet shop staff)	*"Animal welfare office for local council", "Canine massage therapist", "…work inside a pet store"*
Non-canine animal professional (e.g. equine, farm and zoo industry)	*"Equine behaviourist", "Head groom", "Farmer", "Zoo keeper", "Shepherd"*
Miscellaneous free-text response	NONE

Q80: What best describes your current living situation?

- Other (please specify) (n=54)

Back Allocating to Existing Deductive Category	Example(s) Back Allocated
Live alone	*"Live with my other two dogs and two cats, but I am the only human, but do not consider that as living alone.", "I live alone but my dog often goes to my parents house…"*
Live in an adult only home (over 18 years old)	*"Just myself and my partner"*
Live in a home with adults and children	*"2 adults 1 infant", "3 of us - 2 adults and one child"*
Live in a home with children where I am the only adult	NONE

N.B. No new categories were generated as all free-text could be back allocated or was uninterpretable

Q90: Did you use The Puppy Contract when purchasing your puppy? If you did not on this occasion, please explain in your own words why you chose not to or were unable to.

- No (please describe why) (n=803)

Back Allocating to Existing Deductive Category	Example(s) Back Allocated
Yes	NONE

New Category	Example(s)
No, I have a negative perception of The Puppy Contract	*"It was so long", I actually don't like its generic nature and believe that while a contract is necessary this is not the one", "I don't believe they of any value. they certainly aren't worth anything in the eyes of a lawyer", "I did have a contract but I believe the UK template version is woefully inadequate to cover other important factors outside of basic obligations of welfare"*
No, I felt uncomfortable proposing its use and/or feared repercussions from suggesting it	*"Felt a bit awkward asking for this", "My breeder was inexperienced and I feared I would lose the opportunity to buy my puppy if I suggested it.", "My partner felt silly using it so we didn't"*
No, I didn't feel it was needed for the sale of my puppy	*"We had built a healthy relationship with the breeder and continue to keep in contact", "We felt we didn't need to", "We didn't feel it was necessary as we viewed the puppy and picked her up 4 days later"*
No, it was not needed as the breeder is a friend, family member or someone I've bought a puppy from before	*"The puppy came from very close friends…", "The breeder is a very good friend who I trust completely.", "Know the breeder and had previous dog from her"*
I didn't think it was relevant and/or possible for the sale of my puppy	*"Assume it mostly applies to KC registered or council licensed breeders.", "Did not feel all the information in it was necessary, especially for a non pedigree/designer crossbreed…", "Non applicable to me I think"*
No, didn't know enough or feel confident enough about The Puppy Contract to use it	*"Didn't know much about it", "Didn't know enough about it", "Hadn't heard of it until afterwards"*
No, breeder didn't agree to use The Puppy Contract when asked	*"The breeder was not willing", "The breeder wasn't that keen/hadn't heard of it", "The breeder did not want to do that...", "…I asked and he said he was not willing to sign any legal agreement…..'*
No, the breeder didn't offer to use The Puppy Contract (and no indication that the owner asked)	*"My breeder didn't offer it.", "It wasn't offered and I didn't realise I could ask for it", "It was never discussed"*
No, I used a written contract but not The Puppy Contract	*"I used the contract which the breeder provided, which was not the Puppy Contract but one of her own making.", "…The breeder said they have their own contract and did not want to fill out mine…"*
No, I used a verbal contract but not The Puppy Contract	*"…had a verbal contract that he would be returned if he failed a vet check…", "…we verbally agreed if there was a major health issue in the first few weeks I could return her"*
No, I forgot	*"Because I forgot to ask…", "Did not think of it at the time", "Didn't consider it, forgot about it"*
Miscellaneous free-text response	*"Didn't", "No"*
